# Incidence of Neonatal Developmental Dysplasia of the Hip and Late Detection Rates Based on Screening Strategy: A Systematic Review and Meta-analysis

**DOI:** 10.1001/jamanetworkopen.2022.27638

**Published:** 2022-08-18

**Authors:** Ilari Kuitunen, Mikko M. Uimonen, Marjut Haapanen, Reijo Sund, Ilkka Helenius, Ville T. Ponkilainen

**Affiliations:** 1 Institute of Clinical Medicine, Department of Pediatrics, University of Eastern Finland, Kuopio, Finland; 2 Department of Pediatrics and Neonatology, Mikkeli Central Hospital, Mikkeli, Finland; 3 Department of Surgery, Central Finland Hospital Nova, Jyväskylä, Finland; 4 Kuopio Musculoskeletal Research Unit, Institute of Clinical Medicine, University of Eastern Finland, Kuopio, Finland; 5 Department of Orthopaedics and Traumatology, University of Helsinki and Helsinki University Hospital, Helsinki, Finland; 6 Department of Paediatric Orthopedics, Helsinki University Hospital, New Children’s Hospital, Helsinki, Finland

## Abstract

**Importance:**

Universal ultrasonographic screening for developmental dysplasia of the hip (DDH) has gained increasing popularity despite the lack of benefit in terms of reducing the rates of late-detected cases (age ≥12 weeks) in randomized clinical trials.

**Objective:**

To report the reported incidence of DDH in the English scientific literature and compare rates of late-detected cases in settings with different DDH screening strategies.

**Data Sources:**

PubMed, Scopus, and Web of Science databases were searched on November 25 and 27, 2021. No time filters were used in the search.

**Study Selection:**

All observational studies reporting the incidence of early-detected or late-detected (age ≥12 weeks) DDH were included. Non-English reports were excluded if the abstract did not include enough information to be included for analysis.

**Data Extraction and Synthesis:**

The number of newborns screened and the detection rates were extracted. Meta-analysis calculated the pooled incidence of DDH per 1000 newborns with 95% CIs using a random- or fixed-effects model. This study is reported according to the PRISMA and MOOSE guidelines.

**Main Outcomes and Measures:**

The main outcome measures were early detection, early treatment, late detection, and operative treatment incidences.

**Results:**

A total of 1899 studies were identified, 203 full texts were assessed, and 76 studies with 16 901 079 infants were included in final analyses. The early detection rate was 8.4 (95% CI, 4.8-14.8) infants with DDH per 1000 newborns with clinical screening, 4.4 (95% CI, 2.4-8.0) infants with DDH per 1000 newborns with selective ultrasonographic screening, and 23.0 (95% CI, 15.7-33.4) infants with DDH per 1000 newborns with universal ultrasonographic screening. Rates for nonoperative treatment were 5.5 (95% CI, 2.1-14) treatments per 1000 newborns with clinical screening, 3.1 (95% CI, 2.0-4.8) treatments per 1000 newborns with selective ultrasonographic screening, and 9.8 (95% CI, 6.7-14.4) treatments per 1000 newborns with universal ultrasonographic screening. The incidence of late-detected DDH was 0.5 (95% CI, 0.2-1.5) infants with DDH per 1000 newborns with clinical screening, 0.6 (95% CI, 0.3-1.3) infants with DDH per 1000 newborns with selective ultrasonographic screening, and 0.2 (95% CI, 0.0-0.8) infants with DDH per 1000 newborns with universal ultrasonographic screening. The corresponding incidences of operative treatment were 0.2 (95% CI, 0.0-0.9) operations per 1000 newborns with clinical screening, 0.5 (95% CI, 0.4-0.7) operations per 1000 newborns with selective ultrasonographic screening, and 0.4 (95% CI, 0.2-0.7) operations per 1000 newborns with universal ultrasonographic screening.

**Conclusions and Relevance:**

This meta-analysis found that early detection rates and nonoperative treatments were higher with universal screening. The late detection and operative treatment rates with universal screening were similar to those among selectively and clinically screened newborns. Based on these results, universal screening may cause initial overtreatment without reducing the rates of late detection and operative treatment.

## Key Points

**Question:**

Is universal ultrasonographic screening of developmental dysplasia of the hip associated with detecting more patients and reducing the late operative treatment rates compared with selective ultrasonographic and clinical screening?

**Findings:**

In this systematic review and meta-analysis including 76 studies with 16 901 079 patients, the early detection rate in universal ultrasonographic screening was significantly higher than in selective ultrasonographic or clinical screening. However, there were no significant differences in the incidences of late-detected patients and operative treatment among the screening strategies.

**Meaning:**

These findings challenge the rationale for universal ultrasonographic screening, as it was associated with higher initial detection rate without reducing the late detection rate.

## Teaser

This systematic review and meta-analysis reports the incidence of developmental dysplasia of the hip and compares rates of late detection and surgical treatment associated with different screening policies.

## Introduction

Developmental dysplasia of the hip (DDH) includes the following newborn hip findings: “clicky” hip, clinical instability, clinical reversible and irreversible dislocation, immature ultrasonographic findings, and potential dislocation on dynamic ultrasonographic examination.^1,2^ Since Graf^3^ introduced ultrasonographic classification of the hip joint in 1980, the role of ultrasonography in diagnosis and screening has increased.^4,5^ The rationale for this has been that clinical examination does not detect all infants with DDH.^6^ At the moment, universal ultrasonographic screening is especially popular in Central Europe.^7^

The initiation of ultrasonographic screening has led to increased rates of harness and splinting treatments owing to increasing DDH detection.^8^ However, 97% of immature hips detected via ultrasonography normalize during the first months without any treatment.^9^ Observational studies have reported that after the initiation of universal ultrasonographic screening, the rates of late-detected DDH and the need for operative treatment have been lower compared with the prescreening era.^10^ Nonetheless, randomized clinical trials have not found universal screening beneficial or effective in reducing the rate of late-detected DDH.^11,12^ A Cochrane review^13^ in 2013 concluded that the universal screening increases the rate of early treatment but does not reduce the rate of late dislocation. In addition, cost-effectiveness analyses have not recommended the use of universal ultrasonographic screening.^14,15^

Universal screening has been claimed to reduce the need for total hip replacements (THRs) in the future because early treatment may reduce the risk of acetabular dysplasia. However, this has not been widely studied. To our knowledge, there is only 1 large-scale nationwide register study,^16^ conducted in Norway, which did not find any relevant differences in the rates of THR in young adulthood based on the DDH screening status in infancy. The study by Engesaeter et al^16^ was based on clinical screening. Because the optimal DDH screening strategy remains controversial, we aim to report on the incidences of DDH and late-detected DDH and compare the detection and treatment rates among the different screening strategies.

## Methods

We conducted a systematic review and meta-analysis of observational studies reporting the incidence of DDH. We initially searched the literature for new clinical trials systematically but did not find any new randomized trials to add to the most recent Cochrane review^13^; therefore, we decided to focus on observational studies. The search process and search strategy for the randomized clinical trials are presented in eFigure 1 in Supplement 1. The protocol for this study has been sent for registration to PROSPERO (registration ID 300705), but owing to COVID-19–related delays, it has not yet been published. The protocol can also be found in Supplement 2.We report this study according to the Preferred Reporting Items for Systematic Reviews and Meta-analyses (PRISMA) reporting guideline and Meta-analysis of Observational Studies in Epidemiology (MOOSE) reporting guideline.

### Search Strategy

We searched PubMed, Web of Science, and Scopus on November 25 and 27, 2021. We used the following search phrase: (hip) AND (dysplasia) AND (incidence or epidemiolog*) AND (country [eg, Australia or Australian]). The complete screening process is described in eTable 1 in Supplement 1.

We used Covidence systematic review software (Veritas Health Innovation) for the screening process. Each title and abstract was screened independently by 2 of 4 authors participating in screening (I.K., M.M.U., M.H., and V.T.P.). Disagreements were resolved by mutual agreement or third-party opinion by another of the 4 participating authors who did not participate in the study’s initial assessment. Full texts were similarly assessed independently by 2 authors and disagreements were resolved similarly (by mutual agreement or third-party opinion).

### Inclusion Criteria and Main Outcomes and Measures

We included all observational studies (cohort studies, case series, cross-sectional studies, and register-based studies with prospective or retrospective design) that reported the number of patients screened and the event rates for any of the following main outcomes: early-detected DDH (age <12 weeks), nonoperatively treated DDH (ie, splinting or harness treatment), late-detected DDH (age ≥12 weeks), or operatively treated DDH (including closed or open reductions and pelvic or proximal femoral osteotomies).

We excluded studies that did not report the number of patients screened or did not specify the screening method used. We excluded studies focusing only on preterm or breech-delivered newborns or twins. We also excluded non-English reports if all the necessary information was not reported in the abstract in English. The main outcome measures were the country- and screening-stratified incidences of early-detected DDH (age <12 weeks), initial nonoperative treatment rate, incidence of late-detected DDH (age ≥12 weeks), and operative treatment rate.

### Data Extraction

We extracted the data to an Excel 2021 spreadsheet (Microsoft). One author extracted the data (I.K. or V.T.P.), and disagreements were resolved via a second opinion of the other participating author. We chose this extraction method, although it may cause more errors than double data extraction.^17^ We gathered the following information: author, study period, study region, study design, screening method, number of patients screened, screening coverage, number of infants with clinically diagnosed DDH, number of infants with DDH diagnosed via ultrasonography, number of nonoperative treatments, number of operative treatments, and number of infants with late-detected DDH. We stratified the screening process into 3 categories: clinical screening, selective ultrasonographic screening (includes strategies of risk-based ultrasonography and ultrasonography based on clinical findings), and universal ultrasonographic screening. If a study reported incidences prior to and after the implementation of a screening protocol, we included only the after values because this was the current protocol used. The number of dysplastic hips was extracted either per patient or per hip, as reported by the authors. If the study reported only the findings according to Graf classification,^3^ we labeled classes IIb to IV as dysplastic and classes I and IIa as nondysplastic.

### Statistical Analysis

Some of the included studies reported DDH results per hip and not per patient. Thus, we calculated the ratio of dysplastic hips per patient from 7 studies^1,18,19,20,21,22,23^ that included both hips and patients, resulting in a ratio of 1.305 dysplastic hips per patient. The number of hips were multiplied with the ratio to calculate comparable incidences per patient if the number of patients was not presented.

In the meta-analysis, we calculated the pooled incidence of DDH per patient by using a generalized linear mixed model. A random-effects model was used owing to the high heterogeneity (measured with *I*^2^). Incidences are reported per 1000 newborns with 95% CIs. All analyses were performed using R version 4.0.3 (R Project for Statistical Computing), and the pooled incidences were calculated using the function metaprop from the meta package version 5.1-1.

As we included observational studies that reported prevalence data and did not necessarily compared interventions between groups or preintervention vs postintervention periods, risk of bias in nonrandomized studies of interventions (ROBINS-I) was not suitable.^24^ We uses Critical Appraisal Tool for Prevalence Studies by Joanna Briggs Institute.^25^ Statistical differences were determined with overlapping 95% CIs.

## Results

### Included Studies

Our initial search retrieved 1899 results, and we assessed 203 full texts. Ultimately, 76 studies^1,4,5,6,8,18,19,20,21,22,23,26,27,28,29,30,31,32,33,34,35,36,37,38,39,40,41,42,43,44,45,46,47,48,49,50,51,52,53,54,55,56,57,58,59,60,61,62,63,64,65,66,67,68,69,70,71,72,73,74,75,76,77,78,79,80,81,82,83,84,85,86,87,88,89,90^ with 16 901 079 patients were included for analysis (eTable 1 in Supplement 1). Of these studies, 15 concerned clinical screening, 29 concerned selective ultrasound, and 32 concerned universal ultrasound (eTable 2 in Supplement 1). Most studies were conducted in Europe (25 studies [59%]) or Asia (21 studies [28%]). Most of the studies were retrospective (43 studies [57%]) and used single-institute data (60 studies [79%]). Risk of bias was analyzed in 8 domains and overall (eTable 3 in Supplement 1). Most issues were detected in the domains of proper identification of the condition and if the condition was measured similarly to all (eFigure 2 in Supplement 1). None of the studies were excluded based on the risk of bias estimate.

### Incidence of Early-Detected DDH

**Figure 1.**
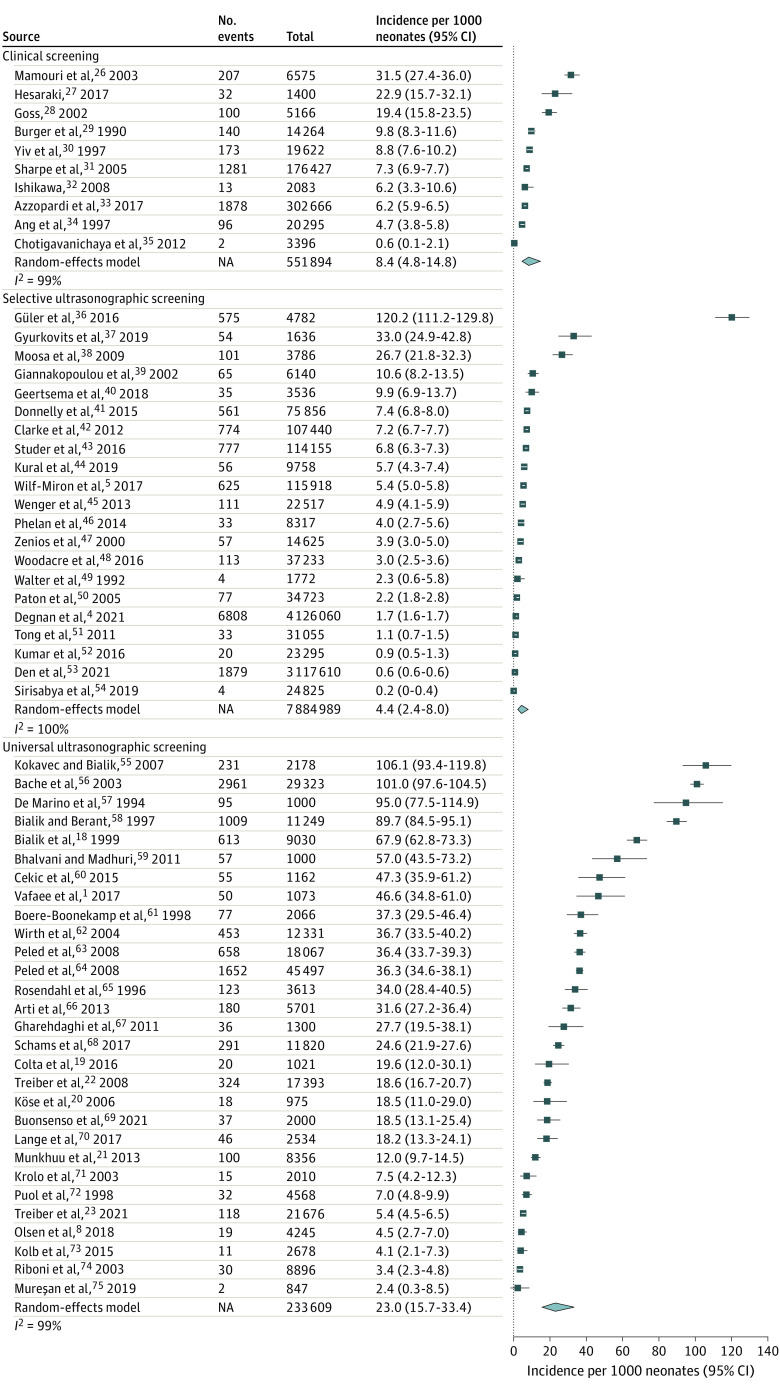
Forest Plot of the Incidence of Early-Detected Developmental Dysplasia of the Hip in Individual Studies Stratified by Screening Strategy A generalized linear random-effects model was used to calculate pooled incidences per 1000 newborns in each screening group. NA indicates not applicable.

**Figure 2.**
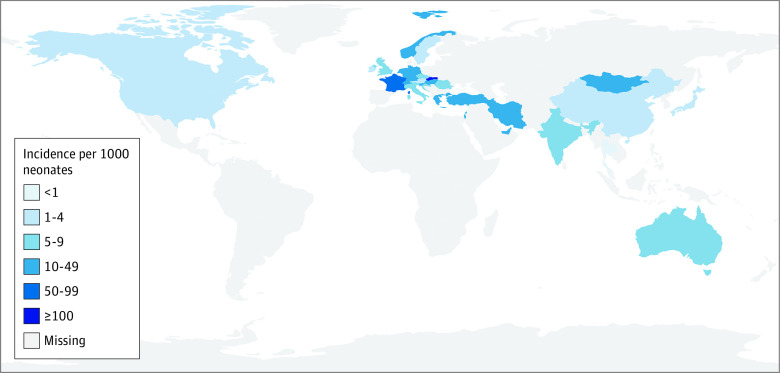
Geographic Variation in the Incidence of Early-Detected Developmental Dysplasia of the Hip Incidences reported per 1000 newborns. Incidence calculated based on either the clinical or ultrasonographic findings per patient, depending on the screening strategy in use.

A total of 60 studies^1,4,5,8,18,19,20,21,22,23,26,27,28,29,30,31,32,33,34,35,36,37,38,39,40,41,42,43,44,45,46,47,48,49,50,51,52,53,54,55,56,57,58,59,60,61,62,63,64,65,66,67,68,69,70,71,72,73,74,75^ including 8 670 492 newborns assessed the incidence of early-detected DDH. Of these, 10 studies^26,27,28,29,30,31,32,33,34,35^ with 551 894 newborns used clinical screening, 21 studies^4,5,36,37,38,39,40,41,42,43,44,45,46,47,48,49,50,51,52,53,54^ with 7 884 989 newborns used selective screening, and 29 studies^1,8,18,19,20,21,22,23,55,56,57,58,59,60,61,62,63,64,65,66,67,68,69,70,71,72,73,74,75^ with 233 609 newborns used universal ultrasonographic screening. The highest reported incidence was 120.3 (95% CI, 110.9-130.5) infants with DDH per 1000 newborns, and the lowest was 0.2 (95% 0.1-0.4) infants with DDH per 1000 newborns (Figure 1). The total pooled incidence estimates were 23.0 (95% CI, 15.7-33.4) infants with DDH per 1000 newborns among those with universal ultrasonographic screening, 4.4 (95% CI, 2.4-8.0) infants with DDH per 1000 newborns among those with selective ultrasonographic screening, and 8.4 (95% CI, 4.8-14.8) infants with DDH per 1000 newborns with clinical screening. The greatest variation was observed among the universally screened populations (Figure 1). Country-specific incidences were highest in Europe (Slovakia and France) and the Middle East (Israel and Iran) (Figure 2). Most countries had not published results in English in indexed peer-reviewed literature regarding the incidence of DDH.

### Incidence of Nonoperative Treatment

**Figure 3.**
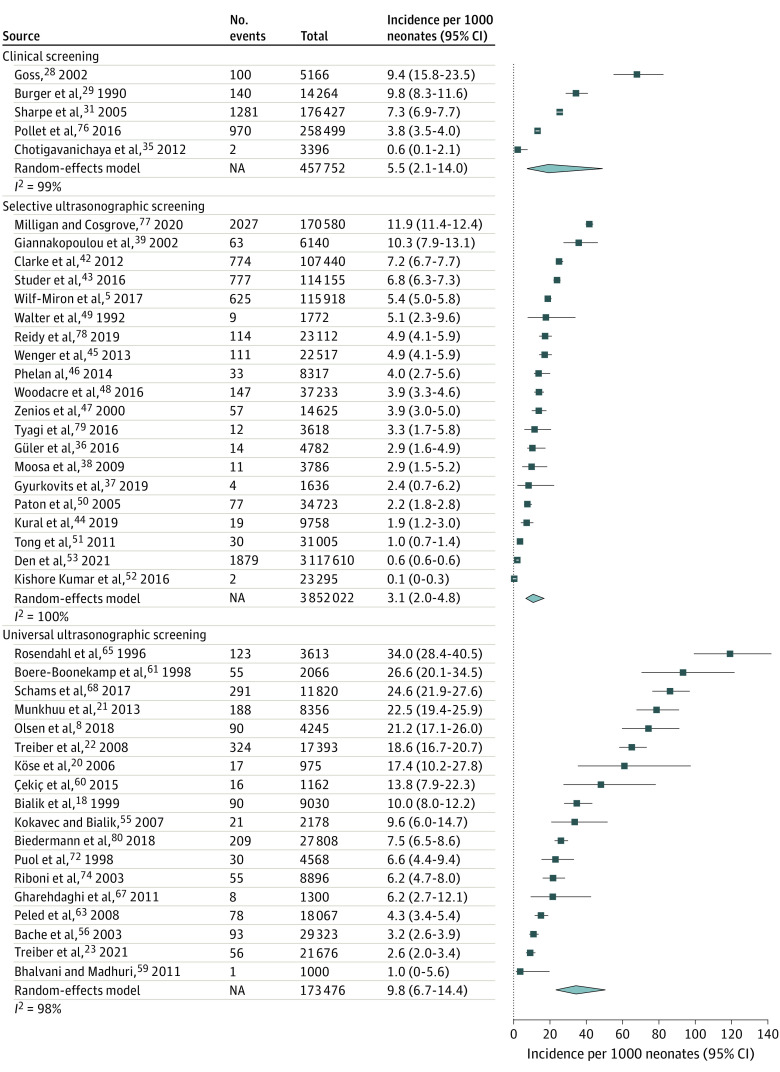
Forest Plot of the Incidence of Initial Nonoperative Treatment for Developmental Dysplasia of the Hip in Individual Studies Stratified by the Screening Strategy Nonoperative treatment included harness or splinting. A random-effects model was used to calculate the pooled incidences per 1000 newborns in each screening group. NA indicates not applicable.

A total of 43 studies^5,8,18,20,21,22,23,28,29,31,35,36,37,38,39,42,43,44,45,46,47,48,49,50,51,52,53,55,56,59,60,61,64,65,67,68,72,74,76,77,78,79,80^ including 4 488 951 newborns assessed the incidence of nonoperatively treated DDH. Of these, 5 studies^28,29,31,35,76^ with 457 752 newborns used clinical screening, 20 studies^5,36,37,38,39,42,43,44,45,46,47,48,49,50,51,52,53,77,78,79^ with 3 852 022 newborns used selective screening, and 18 studies^8,18,20,21,22,23,55,56,59,60,61,64,65,67,68,72,74,80^ with 173 476 newborns used universal ultrasonographic screening. The highest reported incidence was 34.0 (95% CI, 28.5-40.6) treatments per 1000 newborns and the lowest was 0.1 (95% CI, 0.0-0.3) treatments per 1000 newborns (Figure 3). The total pooled incidence estimates were 9.8 (95% CI, 6.7-14.4) treatments per 1000 newborns with universal ultrasonographic screening, 3.1 (95% CI, 2.0-4.8) treatments per 1000 newborns with selective ultrasonographic screening, and 5.4 (95% CI, 2.1-14.0) treatments per 1000 newborns with clinical screening. The greatest variation was observed among the universally screened populations (Figure 1).

### Incidence of Late-Detected DDH

**Figure 4.**
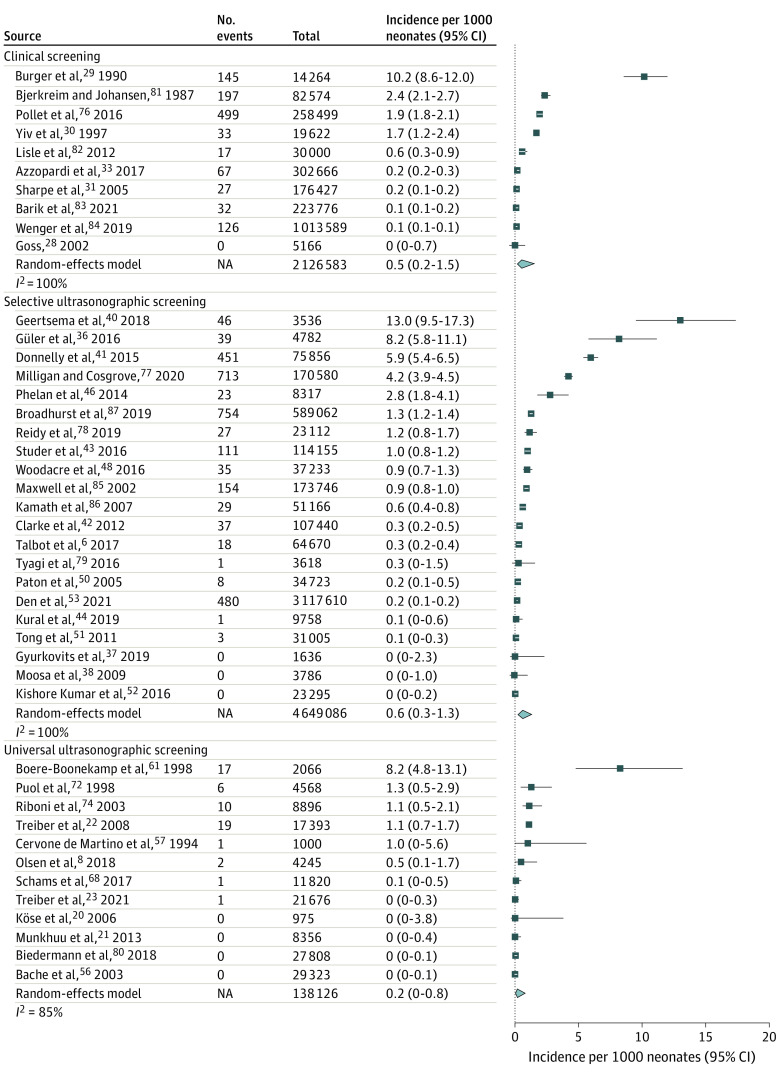
Forest Plot of the Incidence of Late-Detected Developmental Dysplasia of the Hip in Individual Studies Stratified by Screening Strategy Late detection was defined as age 12 weeks or older. A random-effects model was used to calculate pooled incidences per 1000 newborns in each screening group. NA indicates not applicable.

A total of 43 studies^6,8,20,21,22,23,28,29,30,31,33,36,37,38,40,41,42,43,44,46,48,50,51,52,53,56,57,61,68,72,74,76,77,78,79,80,81,82,83,84,85,86,87^ including 6 913 795 newborns assessed the incidence of late-detected DDH. Of these, 10 studies^28,29,30,31,33,76,81,82,83,84^ with 2 126 583 newborns used clinical screening, 21 studies^6,36,37,38,40,41,42,43,44,46,48,50,51,52,53,77,78,79,85,86,87^ with 4 649 086 newborns used selective screening studies, and 12 studies^8,20,21,22,23,56,57,61,68,72,74,80^ with 138 126 newborns used universal ultrasonographic screening. The highest reported incidence was 13.0 (95% CI, 9.7-17.4) infants per 1000 newborns and the lowest was 0.0 (95% CI, 0.0-0.0) infants per 1000 newborns (Figure 3). The total incidence estimates were 0.2 (95% CI, 0.0-0.8) infants per 1000 newborns in universal ultrasonographic screening, 0.6 (95% CI, 0.3-1.3) infants per 1000 newborns in selective ultrasonographic screening, and 0.5 (95% CI, 0.2-1.5) infants per 1000 newborns per 1000 newborns in clinical screening. The greatest variation in incidences was observed among the selectively screened populations (Figure 4).

### Incidence of Operatively Treated DDH

**Figure 5.**
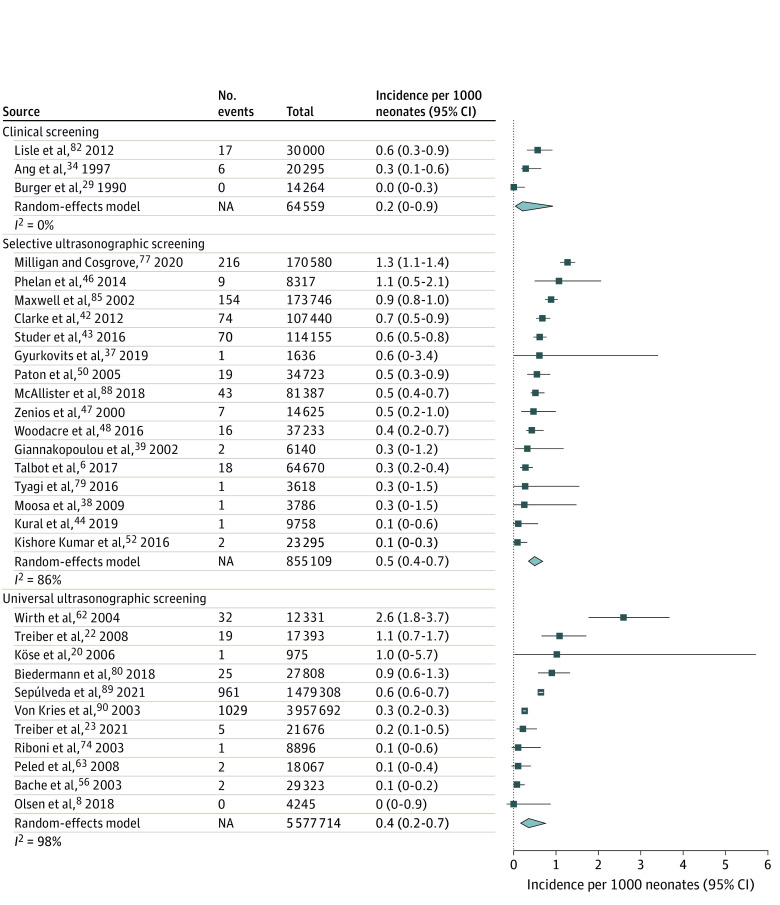
Forest Plot of the Incidence of Operative Treatment of Developmental Dysplasia of the Hip in Individual Studies Stratified by Screening Strategy Operative treatment included closed and open reductions. A random-effects model was used to calculate pooled incidences per 1000 newborns in each screening group. NA indicates not applicable.

A total of 30 studies^6,8,20,22,23,29,34,37,38,39,42,43,44,46,47,48,50,52,56,62,63,74,77,79,80,82,85,88,89^ with 6 497 382 newborns assessed the incidence of operatively treated DDH. Of these, 3 studies^29,34,82^ with 64 559 newborns used clinical screening, 16 studies^6,37,38,39,42,43,44,46,47,48,50,52,77,79,85,88^ with 855 109 newborns used selective screening, and 11 studies^8,20,22,23,56,62,63,74,80,89^ with 5 577 714 newborns used universal ultrasonographic screening. The highest reported incidence was 2.6 (95% CI, 1.8-2.7) operations per 1000 newborns (Figure 3). The total incidence estimates in the random-effects models were 0.4 (95% CI, 0.2-0.7) operations per 1000 newborns with universal ultrasonographic screening, 0.5 (95% CI, 0.4-1.7) operations per 1000 newborns with selective ultrasonographic screening, and 0.2 (95% CI, 0.0-0.9) operations per 1000 newborns with clinical screening (Figure 5).

## Discussion

This systematic review and meta-analysis found that the incidence of DDH detection and nonoperative treatment among newborns given universal ultrasonographic screening was higher than among those given selective or clinical screening. The incidences of late-detected DDH and operatively treated DDH were similar among all screening strategies. The highest DDH burden was found in Europe and the Middle Eastern area of Asia.

Our results are in line with those of previous randomized clinical trials and a meta-analysis of randomized studies.^11,12,13^ However, the use of universal ultrasonographic screening has been increasing, and a recent narrative review stated that, based on the excellent results achieved in observational studies, universal ultrasonography is an effective way to reduce the rate of late-detected DDH^7^; however, this was not seen in our current systematic review and meta-analysis of observational studies.

The early detection rates and nonoperative treatment rates were the lowest in studies with selective ultrasonographic screening. This has previously been shown also in a randomized clinical trial by Elbourne et al,^91^ which found that ultrasonography confirmation of clinically suspected DDH reduced nonoperative treatment rates. However, in the study by Elbourne et al,^91^ the rate of later operative treatment was lower as well, but their follow-up only included patients who were initially referred owing to clinical DDH and did not include patients with late-detected clinical DDH. Thus, based on our findings and the results of the randomized clinical trial performed by Elbourne et al,^91^ it may be that the ultrasonographic confirmation of clinically suspected DDH could reduce the unnecessary nonoperative treatment rate.^91^ The strength of a selective screening policy is that it confirms the clinically suspicious cases of DDH, which could be treated based on the clinical examination findings. Another advantage is that if the selective screening is performed in delayed schedule for patients with hips with clinical suspicion, many of these hips have had time to mature and stabilize.^42^ Studies have shown that most immature hips diagnosed via ultrasonography mature without any interventions during the first weeks of life.^92^ However, it must be noted that in 1 of the largest studies reporting late detection rates before and after selective screening implementation in England,^87^ the rate remained unchanged compared with prior clinical screening policy. The presented late detection rate (1.3 per 1000 neonates) was also extremely high, as studies with clinical screening only have had much lower rates (eg, a study in Sweden^84^ that found a rate of 0.12 per 1000 neonates).

It is obvious that clinical screening will miss a small percentage of patients with DDH (eg, bilateral or teratologic DDH) who could have been treated nonoperatively and will therefore undergo operative treatment at a later stage. The earlier the DDH is detected, the better the outcome, with less complications in early phase and less likely progress to total hip replacement in adolescence or young adulthood.^93,94^ However, the late detection rate and the need for operative treatment are not zero with universal ultrasonographic screening either, because ultrasonography does not detect all patients with clinical instability, and the reproducibility of ultrasonographic findings varies among examiners.^95,96^ For example, nationwide studies from Germany and Austria presented late detected and operated rates for DDH of 0.16 to 0.26 per 1000 neonates, whereas a Swedish study reported a rate of 0.12 per 1000 neonates in clinical screening.^84,90,97^ Therefore, the cost-effectiveness of universal ultrasonographic screening should be critically assessed. Indeed, previous cost-effectiveness studies have stated that universal ultrasonographic screening is not justified based on cost-effectiveness alone but that it could be used in countries with high incidences of DDH to reduce the suffering of a small proportion of patients.^2,14,15,49^ Compared with many globally established cost-effective screening and prevention programs for other diseases (eg, vaccination programs and preterm birth prevention programs) intended to reduce child mortality rates and severe morbidity, given the results of our analysis, universal ultrasonographic screening should be considered carefully and locally, based on the current screening effectiveness and resources. Improvements to clinical screening practices can be made and, for example, swaddling practices can be assessed and considered to be changed prior to implementation of more costly screening programs. The results of our study can be used in decision-making and comparison of acceptable rates of late diagnoses and operative treatments.

Randomized clinical trials are the gold standard in evidence-based medicine.^98^ However, it is not feasible to conduct a randomized clinical trial to address the optimal screening method for DDH owing to the rare event rates. The main outcome measure used to justify universal ultrasonographic screening in the recent literature has been the rates of late-detected DDH and operative treatment. In this meta-analysis, we found that the rate of late-detected DDH was 0.02% among universally screened newborns and 0.05% among clinically or selectively screened newborns, which is not a statistically significant difference. Corresponding operative treatment rates were 0.04% in infants who received universal screening, 0.05% in neonates who received selective ultrasonographic screening, and 0.02% in neonates who received clinical screening, which was also not statistically significant. Therefore, a randomized clinical trial with a 1:1 design comparing a universal screening strategy with a selective strategy would require approximately 61 000 newborns per group to detect the anticipated absolute risk difference of 0.03% in late detection rate (standard α error = .05 and power of 0.80). Given this assumption, the number of screenings needed to avoid a single late-detected DDH would be 3333, and the question of whether this absolute difference should be considered a clinically relevant finding would have to be decided. Considering this, given the clearly underpowered sample sizes, it does not seem surprising that previous randomized clinical trials have not found statistically significant differences, although the late detection rate was high, especially in the study by Rosendahl et al.^11,12^

### Limitations

This study has some limitations. We did not find any new randomized clinical trials since the most recent Cochrane review from 2013,^13^ so we decided not to duplicate previous meta-analysis; therefore, we included only observational studies, which may create a risk of selection and reporting bias in the results. However, based on the risk of bias assessment, the included studies had no major issues. The study settings and reporting practices used in the included studies had high heterogeneity because some of the studies were single-institute prospective series and some of the studies were nationwide register-based retrospective analyses without precise information on the initial hip screening findings. We had to estimate the rates of patients having dysplastic hips because some of the studies reported only pathologic hips in their results, and this may have caused overestimation or underestimation in our results regarding the rates of early-detected DDH. Most studies were conducted in Europe or Asia, and there were no studies from Africa, which creates generalization bias in our global incidence estimations. Furthermore, there are different factors confounding the comparisons between countries; for example, swaddling, clothing for cold weather, and carrying habits have been found to be associated with the incidences of late-detected DDH.^99,100,101,102^ Additionally, we did not search EMBASE database, as it was not available in our institutes, and we also excluded non-English–language reports. Therefore, it is possible that we might have missed some studies. However, our current report is by far the largest effort to gather estimation on the incidences of DDH, late-detected DDH, and non-operative and operative treatments, to our knowledge.

## Conclusions

This systematic review and meta-analysis found that reported rates of early-detected DDH and initial nonoperative treatments are higher in settings with universal ultrasonographic screening compared with clinical screening and selective ultrasonographic screening programs. However, the incidences of late detected DDH and surgical treatment rates were not significantly different among different screening strategies.

## Notes

Supplement 1. **eFigure 1.** Search Strategy and PRISMA Flowchart of the Review Process for Randomized Controlled Trials **eFigure 2.** Overall Risk of Bias Summary Figure for Assessed Domains **eTable 1.** Modified PRISMA Flowchart of the Screening Process **eTable 2.** Background Characteristics of the Included Studies **eTable 3.** Risk of Bias Assessment of the Individual Studies Based on the Joanna Briggs Institute Critical Appraisal Tool for Prevalence Studies

Supplement 2.
Review Protocol

## References

[1] Vafaee AR, Baghdadi T, Baghdadi A, Jamnani RK. DDH epidemiology revisited: do we need new strategies? Arch Bone Jt Surg. 2017;5(6):440-442. 29299500 PMC5736894

[2] Dezateux C, Rosendahl K. Developmental dysplasia of the hip. Lancet. 2007;369(9572):1541-1552. doi:10.1016/S0140-6736(07)60710-7 17482986

[3] Graf R. The diagnosis of congenital hip-joint dislocation by the ultrasonic Combound treatment. Arch Orthop Trauma Surg (1978). 1980;97(2):117-133. doi:10.1007/BF00450934 7458597

[4] Degnan AJ, Hemingway J, Otero HJ, Hughes DR. Developmental hip dysplasia and hip ultrasound frequency in a large American payer database. Clin Imaging. 2021;76:213-216. doi:10.1016/j.clinimag.2021.04.023 33965847

[5] Wilf-Miron R, Kuint J, Peled R, Cohen A, Porath A. Utilization of ultrasonography to detect developmental dysplasia of the hip: when reality turns selective screening into universal use. BMC Pediatr. 2017;17(1):136. doi:10.1186/s12887-017-0882-0 28583152 PMC5460553

[6] Talbot C, Adam J, Paton R. Late presentation of developmental dysplasia of the hip : a 15-year observational study. Bone Joint J. 2017;99-B(9):1250-1255. doi:10.1302/0301-620X.99B9.BJJ-2016-1325.R1 28860408

[7] Kilsdonk I, Witbreuk M, Van Der Woude HJ. Ultrasound of the neonatal hip as a screening tool for DDH: how to screen and differences in screening programs between European countries. J Ultrason. 2021;21(85):e147-e153. doi:10.15557/JoU.2021.0024 34258040 PMC8264809

[8] Olsen SF, Blom HC, Rosendahl K. Introducing universal ultrasound screening for developmental dysplasia of the hip doubled the treatment rate. Acta Paediatr. 2018;107(2):255-261. doi:10.1111/apa.14057 28871598

[9] Rosendahl K, Toma P. Ultrasound in the diagnosis of developmental dysplasia of the hip in newborns—the European approach: a review of methods, accuracy and clinical validity. Eur Radiol. 2007;17(8):1960-1967. doi:10.1007/s00330-006-0557-y 17235535

[10] Biedermann R, Eastwood DM. Universal or selective ultrasound screening for developmental dysplasia of the hip? A discussion of the key issues. J Child Orthop. 2018;12(4):296-301. doi:10.1302/1863-2548.12.180063 30154918 PMC6090188

[11] Rosendahl K, Markestad T, Lie RT. Ultrasound screening for developmental dysplasia of the hip in the neonate: the effect on treatment rate and prevalence of late cases. Pediatrics. 1994;94(1):47-52. 8008537

[12] Holen KJ, Tegnander A, Bredland T, . Universal or selective screening of the neonatal hip using ultrasound: a prospective, randomised trial of 15,529 newborn infants. J Bone Joint Surg Br. 2002;84(6):886-890. doi:10.1302/0301-620X.84B6.0840886 12211684

[13] Shorter D, Hong T, Osborn DA. Cochrane review: screening programmes for developmental dysplasia of the hip in newborn infants. Evid Based Child Health. 2013;8(1):11-54. doi:10.1002/ebch.1891 23878122

[14] Rosendahl K, Markestad T, Lie RT, Sudmann E, Geitung JT. Cost-effectiveness of alternative screening strategies for developmental dysplasia of the hip. Arch Pediatr Adolesc Med. 1995;149(6):643-648. doi:10.1001/archpedi.1995.02170190053009 7767419

[15] Geitung JT, Rosendahl K, Sudmann E. Cost-effectiveness of ultrasonographic screening for congenital hip dysplasia in new-borns. Skeletal Radiol. 1996;25(3):251-254. doi:10.1007/s002560050074 8741062

[16] Engesaeter IØ, Lie SA, Lehmann TG, Furnes O, Vollset SE, Engesaeter LB. Neonatal hip instability and risk of total hip replacement in young adulthood: follow-up of 2,218,596 newborns from the Medical Birth Registry of Norway in the Norwegian Arthroplasty Register. Acta Orthop. 2008;79(3):321-326. doi:10.1080/17453670710015201 18622834

[17] Buscemi N, Hartling L, Vandermeer B, Tjosvold L, Klassen TP. Single data extraction generated more errors than double data extraction in systematic reviews. J Clin Epidemiol. 2006;59(7):697-703. doi:10.1016/j.jclinepi.2005.11.010 16765272

[18] Bialik V, Bialik GM, Blazer S, Sujov P, Wiener F, Berant M. Developmental dysplasia of the hip: a new approach to incidence. Pediatrics. 1999;103(1):93-99. doi:10.1542/peds.103.1.93 9917445

[19] Colta RC, Stoicanescu C, Nicolae M, Oros S, Burnei G. Hip dysplasia screening—epidemiological data from Valcea County. J Med Life. 2016;9(1):106-111. 27489571 PMC4959024

[20] Köse N, Omeroğlu H, Ozyurt B, . Our three-year experience with an ultrasonographic hip screening program conducted in infants at 3 to 4 weeks of age. Article in Turkish. Acta Orthop Traumatol Turc. 2006;40(4):285-290. 17063051

[21] Munkhuu B, Essig S, Renchinnyam E, . Incidence and treatment of developmental hip dysplasia in Mongolia: a prospective cohort study. PLoS One. 2013;8(10):e79427. doi:10.1371/journal.pone.0079427 24205385 PMC3812003

[22] Treiber M, Tomazic T, Tekauc-Golob A, . Ultrasound screening for developmental dysplasia of the hip in the newborn: a population-based study in the Maribor region, 1997-2005. Wien Klin Wochenschr. 2008;120(1-2):31-36. doi:10.1007/s00508-007-0922-0 18239989

[23] Treiber M, Korpar B, Sirše M, Merc M. Early neonatal universal ultrasound screening for developmental dysplasia of the hip: a single institution observational study. Int Orthop. 2021;45(4):991-995. doi:10.1007/s00264-020-04915-0 33459827

[24] Sterne JA, Hernán MA, Reeves BC, . ROBINS-I: a tool for assessing risk of bias in non-randomised studies of interventions. BMJ. 2016;355:i4919. doi:10.1136/bmj.i4919 27733354 PMC5062054

[25] Munn Z, Moola S, Lisy K, Riitano D, Tufanaru C. Methodological guidance for systematic reviews of observational epidemiological studies reporting prevalence and cumulative incidence data. Int J Evid Based Healthc. 2015;13(3):147-153. doi:10.1097/XEB.0000000000000054 26317388

[26] Mamouri GH, Khatami F, Hamedi AB. Congenital dislocation of the hip in newborns in the city of Mashhad. Iran J Med Sci. 2015;28(3):127-130.

[27] Hesaraki M. Investigating the incidence and risk factors for congenital dislocation of the hip joint in infants born in Amirol-Momenin (PBUH) hospital in the city of Zabol in 2016. J Pharm Sci Res. 2017;9(12):2567-2570. Accessed August 3, 2022. https://www.jpsr.pharmainfo.in/Documents/Volumes/vol9Issue12/jpsr09121754.pdf

[28] Goss PW. Successful screening for neonatal hip instability in Australia. J Paediatr Child Health. 2002;38(5):469-474. doi:10.1046/j.1440-1754.2002.00020.x 12354263

[29] Burger BJ, Burger JD, Bos CF, Obermann WR, Rozing PM, Vandenbroucke JP. Neonatal screening and staggered early treatment for congenital dislocation or dysplasia of the hip. Lancet. 1990;336(8730):1549-1553. doi:10.1016/0140-6736(90)93317-I 1979375

[30] Yiv BC, Saidin R, Cundy PJ, . Developmental dysplasia of the hip in South Australia in 1991: prevalence and risk factors. J Paediatr Child Health. 1997;33(2):151-156. doi:10.1111/j.1440-1754.1997.tb01019.x 9145360

[31] Sharpe P, Mulpuri K, Chan A, Cundy PJ. Differences in risk factors between early and late diagnosed developmental dysplasia of the hip. Arch Dis Child Fetal Neonatal Ed. 2006;91(3):F158-F162. doi:10.1136/adc.2004.070870 16332925 PMC2672694

[32] Ishikawa N. The relationship between neonatal developmental dysplasia of the hip and maternal hyperthyroidism. J Pediatr Orthop. 2008;28(4):432-434. doi:10.1097/BPO.0b013e318168d167 18520279

[33] Azzopardi T, Van Essen P, Cundy PJ, Tucker G, Chan A. Late diagnosis of developmental dysplasia of the hip: an analysis of risk factors. J Pediatr Orthop B. 2011;20(1):1-7. doi:10.1097/BPB.0b013e3283415927 21057331

[34] Ang KC, Lee EH, Lee PY, Tan KL. An epidemiological study of developmental dysplasia of the hip in infants in Singapore. Ann Acad Med Singap. 1997;26(4):456-458. 9395810

[35] Chotigavanichaya C, Leurmsumran P, Eamsobhana P, Sanpakit S, Kaewpornsawan K. The incidence of common orthopaedic problems in newborn at Siriraj Hospital. J Med Assoc Thai. 2012;95(suppl 9):S54-S61. 23326983

[36] Güler O, Şeker A, Mutlu S, Çerçi MH, Kömür B, Mahiroğulları M. Results of a universal ultrasonographic hip screening program at a single institution. Acta Orthop Traumatol Turc. 2016;50(1):42-48. doi:10.3944/AOTT.2016.15.0024 26854048

[37] Gyurkovits Z, Sohár G, Baricsa A, Németh G, Orvos H, Dubs B. Early detection of developmental dysplasia of hip by ultrasound. Hip Int. 2021;31(3):424-429. doi:10.1177/1120700019879687 31566007

[38] Moosa NK, Kumar PT, Mahmoodi SM. Incidence of developmental dysplasia of the hip in Dubai. Saudi Med J. 2009;30(7):952-955. 19618014

[39] Giannakopoulou C, Aligizakis A, Korakaki E, . Neonatal screening for developmental dysplasia of the hip on the maternity wards in Crete, Greece. correlation to risk factors. Clin Exp Obstet Gynecol. 2002;29(2):148-152. 12171320

[40] Geertsema D, Meinardi JE, Kempink DRJ, Fiocco M, van de Sande MAJ. Screening program for neonates at risk for developmental dysplasia of the hip: comparing first radiographic evaluation at five months with the standard twelve week ultrasound—a prospective cross-sectional cohort study. Int Orthop. 2019;43(8):1933-1938. doi:10.1007/s00264-018-4089-2 30121837 PMC6647175

[41] Donnelly KJ, Chan KW, Cosgrove AP. Delayed diagnosis of developmental dysplasia of the hip in Northern Ireland: can we do better? Bone Joint J. 2015;97-B(11):1572-1576. doi:10.1302/0301-620X.97B11.35286 26530663

[42] Clarke NMP, Reading IC, Corbin C, Taylor CC, Bochmann T. Twenty years experience of selective secondary ultrasound screening for congenital dislocation of the hip. Arch Dis Child. 2012;97(5):423-429. doi:10.1136/archdischild-2011-301085 22412044

[43] Studer K, Williams N, Antoniou G, . Increase in late diagnosed developmental dysplasia of the hip in South Australia: risk factors, proposed solutions. Med J Aust. 2016;204(6):240. doi:10.5694/mja15.01082 27031400

[44] Kural B, Devecioğlu Karapınar E, Yılmazbaş P, Eren T, Gökçay G. Risk Factor assessment and a ten-year experience of DDH screening in a well-child population. Biomed Res Int. 2019;2019:7213681. doi:10.1155/2019/7213681 31467908 PMC6699317

[45] Wenger D, Düppe H, Tiderius CJ. Acetabular dysplasia at the age of 1 year in children with neonatal instability of the hip. Acta Orthop. 2013;84(5):483-488. doi:10.3109/17453674.2013.850009 24171679 PMC3822134

[46] Phelan N, Thoren J, Fox C, O’Daly BJ, O’Beirne J. Developmental dysplasia of the hip: incidence and treatment outcomes in the Southeast of Ireland. Ir J Med Sci. 2015;184(2):411-415. doi:10.1007/s11845-014-1133-0 24879336

[47] Zenios M, Wilson B, Galasko CS. The effect of selective ultrasound screening on late presenting DDH. J Pediatr Orthop B. 2000;9(4):244-247. doi:10.1097/01202412-200010000-00006 11143466

[48] Woodacre T, Ball T, Cox P. Epidemiology of developmental dysplasia of the hip within the UK: refining the risk factors. J Child Orthop. 2016;10(6):633-642. doi:10.1007/s11832-016-0798-5 27866316 PMC5145848

[49] Walter RS, Donaldson JS, Davis CL, . Ultrasound screening of high-risk infants: a method to increase early detection of congenital dysplasia of the hip. Am J Dis Child. 1992;146(2):230-234. doi:10.1001/archpedi.1992.02160140096028 1733155

[50] Paton RW, Hinduja K, Thomas CD. The significance of at-risk factors in ultrasound surveillance of developmental dysplasia of the hip: a ten-year prospective study. J Bone Joint Surg Br. 2005;87(9):1264-1266. doi:10.1302/0301-620X.87B9.16565 16129755

[51] Tong SHY, Eid MAM, Chow W, To MKT. Screening for developmental dysplasia of the hip in Hong Kong. J Orthop Surg (Hong Kong). 2011;19(2):200-203. doi:10.1177/230949901101900214 21857045

[52] Kishore Kumar R, Shah P, An R, Rajan R. Diagnosing developmental dysplasia of hip in newborns using clinical screen and ultrasound of hips—an Indian experience. J Trop Pediatr. 2016;62(3):241-245. doi:10.1093/tropej/fmv107 26872941

[53] Den H, Ito J, Kokaze A. Epidemiology of developmental dysplasia of the hip: analysis of Japanese national database. J Epidemiol. Published online August 12, 2021. doi:10.2188/jea.JE20210074 34380918 PMC9939923

[54] Sirisabya A, Tooptakong T, Limpaphayom N. Common orthopedic problems in the neonate: a comparative study of 2 periods at a tertiary-care hospital. Asian Biomed. 2019;13(3):101-105. doi:10.1515/abm-2019-0047

[55] Kokavec M, Bialik V. Developmental dysplasia of the hip: prevention and real incidence. Bratisl Lek Listy. 2007;108(6):251-254. 17972535

[56] Bache CE, Clegg J, Herron M. Risk factors for developmental dysplasia of the hip: ultrasonographic findings in the neonatal period. J Pediatr Orthop B. 2002;11(3):212-218. 12089497 10.1097/00009957-200207000-00004

[57] Cervone de Martino M, Riccardi G, Stanzione P, di Lena C, Riccio V. Neonatal screening for congenital hip dislocation: indication of ultrasonography from a systematic study correlating clinical findings and ultrasonography. Article in French. Rev Chir Orthop Reparatrice Appar Mot. 1994;80(4):320-323. 7740133

[58] Bialik V, Berant M. “Immunity” of Ethiopian Jews to developmental dysplasia of the hip: a preliminary sonographic study. J Pediatr Orthop B. 1997;6(4):253-254. doi:10.1097/01202412-199710000-00006 9343784

[59] Bhalvani C, Madhuri V. Ultrasound profile of hips of South Indian infants. Indian Pediatr. 2011;48(6):475-477. doi:10.1007/s13312-011-0075-0 21555794

[60] Çekiç B, Erdem-Toslak İ, Sertkaya Ö, . Incidence and follow-up outcomes of developmental hip dysplasia of newborns in the Western Mediterranean Region. Turk J Pediatr. 2015;57(4):353-358. 27186697

[61] Boere-Boonekamp MM, Kerkhoff TH, Schuil PB, Zielhuis GA. Early detection of developmental dysplasia of the hip in the Netherlands: the validity of a standardized assessment protocol in infants. Am J Public Health. 1998;88(2):285-288. doi:10.2105/AJPH.88.2.285 9491024 PMC1508179

[62] Wirth T, Stratmann L, Hinrichs F. Evolution of late presenting developmental dysplasia of the hip and associated surgical procedures after 14 years of neonatal ultrasound screening. J Bone Joint Surg Br. 2004;86(4):585-589. doi:10.1302/0301-620X.86B4.14586 15174558

[63] Peled E, Bialik V, Katzman A, Eidelman M, Norman D. Treatment of Graf’s ultrasound class III and IV hips using Pavlik’s method. Clin Orthop Relat Res. 2008;466(4):825-829. doi:10.1007/s11999-008-0119-5 18288557 PMC2504669

[64] Peled E, Eidelman M, Katzman A, Bialik V. Neonatal incidence of hip dysplasia: ten years of experience. Clin Orthop Relat Res. 2008;466(4):771-775. doi:10.1007/s11999-008-0132-8 18288551 PMC2504674

[65] Rosendahl K, Markestad T, Lie RT. Developmental dysplasia of the hip: a population-based comparison of ultrasound and clinical findings. Acta Paediatr. 1996;85(1):64-69. doi:10.1111/j.1651-2227.1996.tb13892.x 8834982

[66] Arti H, Mehdinasab SA, Arti S. Comparing results of clinical versus ultrasonographic examination in developmental dysplasia of hip. J Res Med Sci. 2013;18(12):1051-1055. 24523795 PMC3908525

[67] Gharedaghi M, Mohammadzadeh A, Zandi B. Comparison of clinical and sonographic prevalence of developmental dysplasia of the hip. Acta Med Iran. 2011;49(1):25-27. 21425067

[68] Schams M, Labruyère R, Zuse A, Walensi M. Diagnosing developmental dysplasia of the hip using the Graf ultrasound method: risk and protective factor analysis in 11,820 universally screened newborns. Eur J Pediatr. 2017;176(9):1193-1200. doi:10.1007/s00431-017-2959-z 28717864

[69] Buonsenso D, Curatola A, Lazzareschi I, . Developmental dysplasia of the hip: real world data from a retrospective analysis to evaluate the effectiveness of universal screening. J Ultrasound. 2021;24(4):403-410. doi:10.1007/s40477-020-00463-w 32356221 PMC8572248

[70] Lange AE, Lange J, Ittermann T, . Population-based study of the incidence of congenital hip dysplasia in preterm infants from the Survey of Neonates in Pomerania (SNIP). BMC Pediatr. 2017;17(1):78. doi:10.1186/s12887-017-0829-5 28302080 PMC5356283

[71] Krolo I, Visković K, Kozić S, . The advancement in the early diagnostics of developmental hip dysplasia in infants—the role of ultrasound screening. Coll Antropol. 2003;27(2):627-634. 14746152

[72] Poul J, Bajerová J, Skotáková J, Jíra I. Selective treatment program for developmental dysplasia of the hip in an epidemiologic prospective study. J Pediatr Orthop B. 1998;7(2):135-137. doi:10.1097/01202412-199804000-00008 9597589

[73] Kolb A, Schweiger N, Mailath-Pokorny M, . Low incidence of early developmental dysplasia of the hip in universal ultrasonographic screening of newborns: analysis and evaluation of risk factors. Int Orthop. 2016;40(1):123-127. doi:10.1007/s00264-015-2799-2 25940606

[74] Riboni G, Bellini A, Serantoni S, Rognoni E, Bisanti L. Ultrasound screening for developmental dysplasia of the hip. Pediatr Radiol. 2003;33(7):475-481. doi:10.1007/s00247-003-0940-7 12750862

[75] Mureşan S, Mărginean MO, Voidăzan S, Vlasa I, Sîntean I. Musculoskeletal ultrasound: a useful tool for diagnosis of hip developmental dysplasia: one single-center experience. Medicine (Baltimore). 2019;98(2):e14081. doi:10.1097/MD.0000000000014081 30633215 PMC6336624

[76] Pollet V, Percy V, Prior HJ. Relative risk and incidence for developmental dysplasia of the hip. J Pediatr. 2017;181:202-207. doi:10.1016/j.jpeds.2016.10.017 27866823

[77] Milligan DJ, Cosgrove AP. Monitoring of a hip surveillance programme protects infants from radiation and surgical intervention. Bone Joint J. 2020;102-B(4):495-500. doi:10.1302/0301-620X.102B4.BJJ-2019-0809.R2 32228072 PMC7136684

[78] Reidy M, Collins C, MacLean JGB, Campbell D. Examining the effectiveness of examination at 6-8 weeks for developmental dysplasia: testing the safety net. Arch Dis Child. 2019;104(10):953-955. doi:10.1136/archdischild-2018-316520 30518519

[79] Tyagi R, Zgoda MR, Short R. Targeted screening of hip dysplasia in newborns: experience at a district general hospital in Scotland. Orthop Rev (Pavia). 2016;8(3):6640. doi:10.4081/or.2016.6640 27761220 PMC5066110

[80] Biedermann R, Riccabona J, Giesinger JM, . Results of universal ultrasound screening for developmental dysplasia of the hip: a prospective follow-up of 28 092 consecutive infants. Bone Joint J. 2018;100-B(10):1399-1404. doi:10.1302/0301-620X.100B10.BJJ-2017-1539.R2 30295526

[81] Bjerkreim I, Johansen J. Late diagnosed congenital dislocation of the hip. Acta Orthop Scand. 1987;58(5):504-506. doi:10.3109/17453678709146388 3425278

[82] Lisle R, Boekelaar M, Stannage K, Whitewood C. Delayed diagnosis of developmental dislocation of the hip: the Western Australian experience. ANZ J Surg. 2012;82(9):612-615. doi:10.1111/j.1445-2197.2012.06110.x 22889248

[83] Barik S, Pandita N, Paul S, Kumari O, Singh V. Prevalence of congenital limb defects in Uttarakhand state in India—a hospital-based retrospective cross-sectional study. Clin Epidemiol Glob Health. 2021;9:99-103. doi:10.1016/j.cegh.2020.07.007

[84] Wenger D, Düppe H, Nilsson JÅ, Tiderius CJ. Incidence of late-diagnosed hip dislocation after universal clinical screening in Sweden. JAMA Netw Open. 2019;2(11):e1914779. doi:10.1001/jamanetworkopen.2019.14779 31702798 PMC6902841

[85] Maxwell SL, Ruiz AL, Lappin KJ, Cosgrove AP. Clinical screening for developmental dysplasia of the hip in Northern Ireland. BMJ. 2002;324(7344):1031-1033. doi:10.1136/bmj.324.7344.1031 11976249 PMC1122960

[86] Kamath S, Mehdi A, Wilson N, Duncan R. The lack of evidence of the effect of selective ultrasound screening on the incidence of late developmental dysplasia of the hip in the Greater Glasgow Region. J Pediatr Orthop B. 2007;16(3):189-191. doi:10.1097/01.bpb.0000236229.44819.43 17414779

[87] Broadhurst C, Rhodes AML, Harper P, Perry DC, Clarke NMP, Aarvold A. What is the incidence of late detection of developmental dysplasia of the hip in England: a 26-year national study of children diagnosed after the age of one. Bone Joint J. 2019;101-B(3):281-287. doi:10.1302/0301-620X.101B3.BJJ-2018-1331.R1 30813797

[88] McAllister DA, Morling JR, Fischbacher CM, Reidy M, Murray A, Wood R. Enhanced detection services for developmental dysplasia of the hip in Scottish children, 1997-2013. Arch Dis Child. 2018;103(11):1021-1026. doi:10.1136/archdischild-2017-314354 29436408 PMC6225802

[89] Sepúlveda MF, Pérez JA, Saban EA, Castañeda LE, Sepúlveda DF, Birrer EAM. Developmental dysplasia of the hip screening programme in Chile. J Child Orthop. 2021;15(1):35-41. doi:10.1302/1863-2548.15.200240 33643456 PMC7907761

[90] von Kries R, Ihme N, Oberle D, . Effect of ultrasound screening on the rate of first operative procedures for developmental hip dysplasia in Germany. Lancet. 2003;362(9399):1883-1887. doi:10.1016/S0140-6736(03)14957-4 14667743

[91] Elbourne D, Dezateux C, Arthur R, ; UK Collaborative Hip Trial Group. Ultrasonography in the diagnosis and management of developmental hip dysplasia (UK Hip Trial): clinical and economic results of a multicentre randomised controlled trial. Lancet. 2002;360(9350):2009-2017. doi:10.1016/S0140-6736(02)12024-1 12504396

[92] Liu B, Hu X, Li L, Gao S. Morphological development of the hip in normal infants under six months of age by the Graf ultrasound method. Front Pediatr. 2022;10:914545. doi:10.3389/fped.2022.914545 35615629 PMC9126495

[93] Terjesen T, Horn J, Gunderson RB. Fifty-year follow-up of late-detected hip dislocation: clinical and radiographic outcomes for seventy-one patients treated with traction to obtain gradual closed reduction. J Bone Joint Surg Am. 2014;96(4):e28. doi:10.2106/JBJS.M.00397 24553897

[94] Albinana J, Dolan LA, Spratt KF, Morcuende J, Meyer MD, Weinstein SL. Acetabular dysplasia after treatment for developmental dysplasia of the hip: implications for secondary procedures. J Bone Joint Surg Br. 2004;86(6):876-886. doi:10.1302/0301-620X.86B6.14441 15330030

[95] Chang WC, Hsu KH, Lo IF, Liao KH, Su YP. Interobserver agreement and clinical disparity between the Graf method and femoral head coverage measurement in developmental dysplasia of the hip screening: a prospective observational study of 198 newborns. Medicine (Baltimore). 2021;100(24):e26291. doi:10.1097/MD.0000000000026291 34128864 PMC8213240

[96] Ghasseminia S, Hareendranathan AR, Jaremko JL. Narrative review on the role of imaging in DDH. Indian J Orthop. 2021;55(6):1456-1465. doi:10.1007/s43465-021-00511-5 35003536 PMC8688667

[97] Thallinger C, Pospischill R, Ganger R, Radler C, Krall C, Grill F. Long-term results of a nationwide general ultrasound screening system for developmental disorders of the hip: the Austrian hip screening program. J Child Orthop. 2014;8(1):3-10. doi:10.1007/s11832-014-0555-6 24488847 PMC3935031

[98] Djulbegovic B, Guyatt GH. Progress in evidence-based medicine: a quarter century on. Lancet. 2017;390(10092):415-423. doi:10.1016/S0140-6736(16)31592-6 28215660

[99] Loder RT, Shafer C. Seasonal variation in children with developmental dysplasia of the hip. J Child Orthop. 2014;8(1):11-22. doi:10.1007/s11832-014-0558-3 24500336 PMC3935022

[100] Ulziibat M, Munkhuu B, Bataa AE, Schmid R, Baumann T, Essig S. Traditional Mongolian swaddling and developmental dysplasia of the hip: a randomized controlled trial. BMC Pediatr. 2021;21:450. doi:10.1186/s12887-021-02910-x 34641800 PMC8513275

[101] Lee WC, Kao HK, Wang SM, Yang WE, Chang CH, Kuo KN. Cold weather as a risk factor for late diagnosis and surgery for developmental dysplasia of the hip. J Bone Joint Surg Am. 2022;104(2):115-122. doi:10.2106/JBJS.21.00460 34793368

[102] Graham SM, Manara J, Chokotho L, Harrison WJ. Back-carrying infants to prevent developmental hip dysplasia and its sequelae: is a new public health initiative needed? J Pediatr Orthop. 2015;35(1):57-61. doi:10.1097/BPO.0000000000000234 24942071

