# Supplementary material for: Incidence of Neonatal Developmental Dysplasia of the Hip and Late Detection Rates Based on Screening Strategy: A Systematic Review and Meta-analysis
Source: JAMA Netw Open. 2022 Aug 18;5(8):e2227638. doi: 10.1001/jamanetworkopen.2022.27638 (PMC9389349; doi:10.1001/jamanetworkopen.2022.27638)
Supplement: Supplement 1. — eFigure 1. Search Strategy and PRISMA Flowchart of the Review Process for Randomized Controlled Trials eFigure 2. Overall Risk of Bias Summary Figure for Assessed Domains eTable 1. Modified PRISMA Flowchart of the Screening Process eTable 2. Background Characteristics of the Included Studies eTable 3. Risk of Bias Assessment of the Individual Studies Based on the Joanna Briggs Institute Critical Appraisal Tool for Prevalence Studies [file jamanetwopen-e2227638-s001.pdf]

Supplemental Online Content

Kuitunen I, Uimonen MM, Haapanen M, Sund R, Helenius I, Ponkilainen VT. Incidence of neonatal developmental dysplasia of the hip and late detection rates based on screening strategy: a systematic review and meta-analysis. *JAMA Netw Open*. 2022;5(8):e2227638. doi:10.1001/jamanetworkopen.2022.27638

eFigure 1. Search Strategy and PRISMA Flowchart of the Review Process for Randomized Controlled Trials

eFigure 2. Overall Risk of Bias Summary Figure for Assessed Domains

eTable 1. Modified PRISMA Flowchart of the Screening Process

eTable 2. Background Characteristics of the Included Studies

eTable 3. Risk of Bias Assessment of the Individual Studies Based on the Joanna Briggs Institute Critical Appraisal Tool for Prevalence Studies

This supplemental material has been provided by the authors to give readers additional information about their work.

© 2022 Kuitunen I et al. *JAMA Network Open.*

eFigure 1. Search Strategy and PRISMA Flowchart of the Review Process for Randomized Controlled Trials

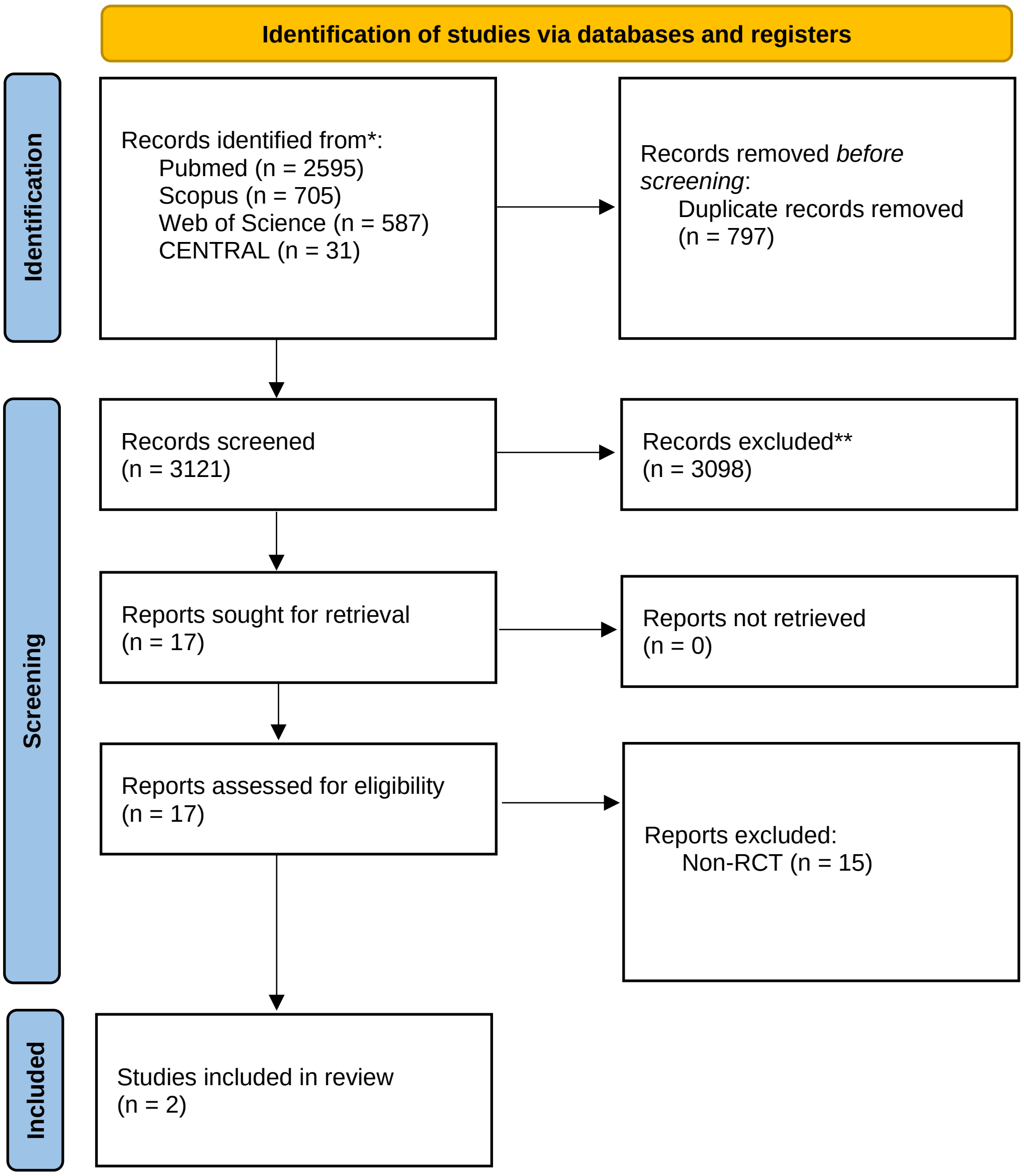

As none of these records were novel to the Cochrane review in 2013, we decided not to conduct synthesis of these studies as replication was not needed.

Search strategy:

Following search term was used: (hip) AND (ultrasound) AND (luxation or dysplasia) AND (newborn or neonate or congenital)
Following databases were searched: PubMed, Scopus, Web of Science, CENTRAL.
Date of search: November 16th, 2021.

© 2022 Kuitunen I et al. *JAMA Network Open.*

eFigure 2. Overall Risk of Bias Summary Figure for Assessed Domains

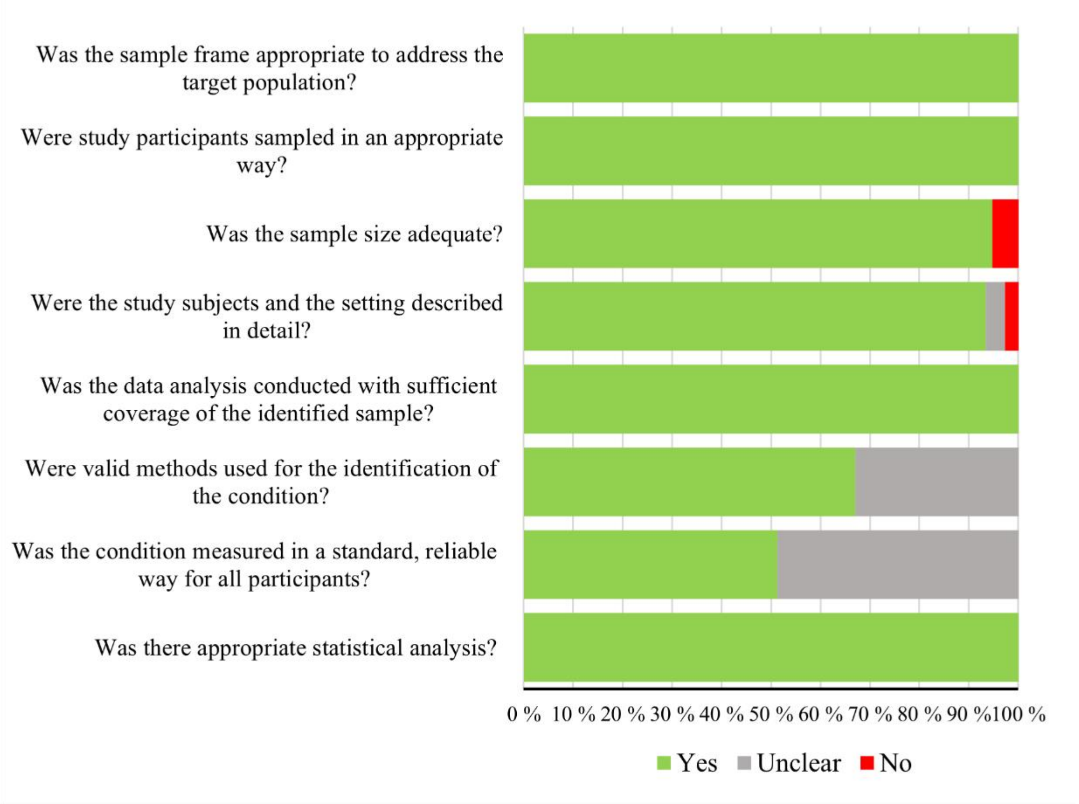

© 2022 Kuitunen I et al. *JAMA Network Open.*

eTable 1. Modified PRISMA Flowchart of the Screening Process

#	Country	Date of search	Results retrieved	Duplicates removed	Abstracts screened	Records excluded	Full texts assessed	Reports excluded	Studies included for review
1	Afghanistan	25.11.2021	0	0	0	0	0	0	0
2	Albania	25.11.2021	0	0	0	0	0	0	0
3	Algeria	25.11.2021	1	0	1	1	0	0	0
4	Andorra	25.11.2021	0	0	0	0	0	0	0
5	Angola	25.11.2021	0	0	0	0	0	0	0
6	Antigua and Barbuda	25.11.2021	0	0	0	0	0	0	0
7	Argentina	25.11.2021	3	0	3	3	0	0	0
8	Armenia	25.11.2021	0	0	0	0	0	0	0
9	Australia	25.11.2021	51	7	44	37	7	1	6
10	Austria	25.11.2021	16	1	15	8	7	5	2
11	Azerbaijan	25.11.2021	0	0	0	0	0	0	0
12	Bahamas	25.11.2021	0	0	0	0	0	0	0
13	Bahrain	25.11.2021	0	0	0	0	0	0	0
14	Bangladesh	25.11.2021	2	1	1	1	0	0	0
15	Barbados	25.11.2021	0	0	0	0	0	0	0
16	Belarus	25.11.2021	0	0	0	0	0	0	0
17	Belgium	25.11.2021	12	1	11	11	0	0	0
18	Belize	25.11.2021	0	0	0	0	0	0	0
19	Benin	25.11.2021	0	0	0	0	0	0	0
20	Bhutan	25.11.2021	0	0	0	0	0	0	0
21	Bolivia	25.11.2021	0	0	0	0	0	0	0
22	Bosnia and Herzegovina	25.11.2021	2	0	2	1	1	0	1

© 2022 Kuitunen I et al. *JAMA Network Open.*

**23**	**Botswana**	**25.11. 2021**	**0**	**0**	**0**	**0**	**0**	**0**	**0**
**24**	**Brazil**	**25.11. 2021**	**14**	**1**	**13**	**13**	**0**	**0**	**0**
**25**	**Brunei**	**25.11. 2021**	**0**	**0**	**0**	**0**	**0**	**0**	**0**
**26**	**Bulgaria**	**25.11. 2021**	**2**	**0**	**2**	**1**	**1**	**1**	**0**
**27**	**Burkina Faso**	**25.11. 2021**	**2**	**1**	**1**	**1**	**0**	**0**	**0**
**28**	**Burundi**	**25.11. 2021**	**0**	**0**	**0**	**0**	**0**	**0**	**0**
**29**	**Côte d'Ivoire**	**25.11. 2021**	**0**	**0**	**0**	**0**	**0**	**0**	**0**
**30**	**Cabo Verde**	**25.11. 2021**	**0**	**0**	**0**	**0**	**0**	**0**	**0**
**31**	**Cambodia**	**25.11. 2021**	**0**	**0**	**0**	**0**	**0**	**0**	**0**
**32**	**Cameroon**	**25.11. 2021**	**0**	**0**	**0**	**0**	**0**	**0**	**0**
**33**	**Canada**	**25.11. 2021**	**34**	**2**	**32**	**31**	**1**	**0**	**1**
**34**	**Central African Republic**	**25.11. 2021**	**0**	**0**	**0**	**0**	**0**	**0**	**0**
**35**	**Chad**	**25.11. 2021**	**0**	**0**	**0**	**0**	**0**	**0**	**0**
**36**	**Chile**	**25.11. 2021**	**7**	**2**	**5**	**4**	**1**	**0**	**1**
**37**	**China**	**25.11. 2021**	**121**	**7**	**114**	**110**	**4**	**3**	**1**
**38**	**Colombia**	**25.11. 2021**	**3**	**1**	**2**	**2**	**0**	**0**	**0**
**39**	**Comoros**	**25.11. 2021**	**0**	**0**	**0**	**0**	**0**	**0**	**0**
**40**	**Congo (Congo-Brazzaville)**	**25.11. 2021**	**0**	**0**	**0**	**0**	**0**	**0**	**0**
**41**	**Costa Rica**	**25.11. 2021**	**0**	**0**	**0**	**0**	**0**	**0**	**0**
**42**	**Croatia**	**25.11. 2021**	**10**	**1**	**9**	**5**	**4**	**4**	**0**
**43**	**Cuba**	**25.11. 2021**	**0**	**0**	**0**	**0**	**0**	**0**	**0**
**44**	**Cyprus**	**25.11. 2021**	**0**	**0**	**0**	**0**	**0**	**0**	**0**
**45**	**Czechia (Czech Republic)**	**25.11. 2021**	**17**	**1**	**16**	**11**	**5**	**4**	**1**
**46**	**Democratic Republic of the Congo**	**25.11. 2021**	**0**	**0**	**0**	**0**	**0**	**0**	**0**
**47**	**Denmark**	**25.11. 2021**	**37**	**6**	**31**	**28**	**3**	**3**	**0**
**48**	**Djibouti**	**25.11. 2021**	**0**	**0**	**0**	**0**	**0**	**0**	**0**

**49**	**Dominica**	**25.11. 2021**	**0**	**0**	**0**	**0**	**0**	**0**	**0**
**50**	**Dominican Republic**	**25.11. 2021**	**0**	**0**	**0**	**0**	**0**	**0**	**0**
**51**	**Ecuador**	**25.11. 2021**	**0**	**0**	**0**	**0**	**0**	**0**	**0**
**52**	**Egypt**	**25.11. 2021**	**5**	**2**	**3**	**3**	**0**	**0**	**0**
**53**	**El Salvador**	**25.11. 2021**	**0**	**0**	**0**	**0**	**0**	**0**	**0**
**54**	**Equatorial Guinea**	**25.11. 2021**	**0**	**0**	**0**	**0**	**0**	**0**	**0**
**55**	**Eritrea**	**25.11. 2021**	**0**	**0**	**0**	**0**	**0**	**0**	**0**
**56**	**Estonia**	**25.11. 2021**	**0**	**0**	**0**	**0**	**0**	**0**	**0**
**57**	**Eswatini (fmr. "Swaziland")**	**25.11. 2021**	**0**	**0**	**0**	**0**	**0**	**0**	**0**
**58**	**Ethiopia**	**25.11. 2021**	**9**	**3**	**6**	**3**	**3**	**1**	**2**
**59**	**Fiji**	**25.11. 2021**	**0**	**0**	**0**	**0**	**0**	**0**	**0**
**60**	**Finland**	**25.11. 2021**	**11**	**1**	**10**	**10**	**0**	**0**	**0**
**61**	**France**	**25.11. 2021**	**51**	**5**	**46**	**42**	**4**	**3**	**1**
**62**	**Gabon**	**26.11. 2021**	**0**	**0**	**0**	**0**	**0**	**0**	**0**
**63**	**Gambia**	**26.11. 2021**	**0**	**0**	**0**	**0**	**0**	**0**	**0**
**64**	**Georgia**	**26.11. 2021**	**0**	**0**	**0**	**0**	**0**	**0**	**0**
**65**	**Germany**	**26.11. 2021**	**144**	**18**	**126**	**105**	**21**	**19**	**2**
**66**	**Ghana**	**26.11. 2021**	**0**	**0**	**0**	**0**	**0**	**0**	**0**
**67**	**Greece**	**26.11. 2021**	**6**	**1**	**5**	**4**	**1**	**0**	**1**
**68**	**Grenada**	**26.11. 2021**	**2**	**1**	**1**	**1**	**0**	**0**	**0**
**69**	**Guatemala**	**26.11. 2021**	**2**	**1**	**1**	**1**	**0**	**0**	**0**
**70**	**Guinea**	**26.11. 2021**	**0**	**0**	**0**	**0**	**0**	**0**	**0**
**71**	**Guinea-Bissau**	**26.11. 2021**	**0**	**0**	**0**	**0**	**0**	**0**	**0**
**72**	**Guyana**	**26.11. 2021**	**0**	**0**	**0**	**0**	**0**	**0**	**0**
**73**	**Haiti**	**26.11. 2021**	**0**	**0**	**0**	**0**	**0**	**0**	**0**
**74**	**Holy See**	**26.11. 2021**	**0**	**0**	**0**	**0**	**0**	**0**	**0**
**75**	**Honduras**	**26.11. 2021**	**0**	**0**	**0**	**0**	**0**	**0**	**0**

© 2022 Kuitunen I et al. *JAMA Network Open.*

**76**	**Hungary**	**26.11. 2021**	**15**	**2**	**13**	**10**	**3**	**2**	**1**
**77**	**Iceland**	**26.11. 2021**	**0**	**0**	**0**	**0**	**0**	**0**	**0**
**78**	**India**	**26.11. 2021**	**36**	**5**	**31**	**26**	**5**	**2**	**3**
**79**	**Indonesia**	**26.11. 2021**	**0**	**0**	**0**	**0**	**0**	**0**	**0**
**80**	**Iran**	**26.11. 2021**	**11**	**1**	**10**	**5**	**5**	**0**	**5**
**81**	**Iraq**	**26.11. 2021**	**2**	**0**	**2**	**2**	**0**	**0**	**0**
**82**	**Ireland**	**26.11. 2021**	**39**	**6**	**33**	**22**	**11**	**7**	**4**
**83**	**Israel**	**26.11. 2021**	**15**	**2**	**13**	**7**	**6**	**3**	**3**
**84**	**Italy**	**26.11. 2021**	**45**	**5**	**40**	**27**	**13**	**11**	**2**
**85**	**Jamaica**	**26.11. 2021**	**0**	**0**	**0**	**0**	**0**	**0**	**0**
**86**	**Japan**	**26.11. 2021**	**132**	**27**	**105**	**101**	**4**	**2**	**2**
**87**	**Jordan**	**26.11. 2021**	**5**	**0**	**5**	**4**	**1**	**1**	**0**
**88**	**Kazakhstan**	**26.11. 2021**	**0**	**0**	**0**	**0**	**0**	**0**	**0**
**89**	**Kenya**	**26.11. 2021**	**0**	**0**	**0**	**0**	**0**	**0**	**0**
**90**	**Kiribati**	**26.11. 2021**	**0**	**0**	**0**	**0**	**0**	**0**	**0**
**91**	**Kuwait**	**26.11. 2021**	**2**	**1**	**1**	**1**	**0**	**0**	**0**
**92**	**Kyrgyzstan**	**26.11. 2021**	**0**	**0**	**0**	**0**	**0**	**0**	**0**
**93**	**Laos**	**26.11. 2021**	**0**	**0**	**0**	**0**	**0**	**0**	**0**
**94**	**Latvia**	**26.11. 2021**	**0**	**0**	**0**	**0**	**0**	**0**	**0**
**95**	**Lebanon**	**26.11. 2021**	**4**	**0**	**4**	**4**	**0**	**0**	**0**
**96**	**Lesotho**	**26.11. 2021**	**0**	**0**	**0**	**0**	**0**	**0**	**0**
**97**	**Liberia**	**26.11. 2021**	**0**	**0**	**0**	**0**	**0**	**0**	**0**
**98**	**Libya**	**26.11. 2021**	**0**	**0**	**0**	**0**	**0**	**0**	**0**
**99**	**Liechtenstein**	**26.11. 2021**	**0**	**0**	**0**	**0**	**0**	**0**	**0**
**100**	**Lithuania**	**26.11. 2021**	**0**	**0**	**0**	**0**	**0**	**0**	**0**
**101**	**Luxembourg**	**26.11. 2021**	**0**	**0**	**0**	**0**	**0**	**0**	**0**
**102**	**Madagascar**	**26.11. 2021**	**0**	**0**	**0**	**0**	**0**	**0**	**0**

© 2022 Kuitunen I et al. *JAMA Network Open.*

**103**	**Malawi**	**26.11. 2021**	**3**	**1**	**2**	**1**	**1**	**1**	**0**
**104**	**Malaysia**	**26.11. 2021**	**5**	**1**	**4**	**1**	**3**	**2**	**1**
**105**	**Maldives**	**26.11. 2021**	**0**	**0**	**0**	**0**	**0**	**0**	**0**
**106**	**Mali**	**26.11. 2021**	**0**	**0**	**0**	**0**	**0**	**0**	**0**
**107**	**Malta**	**26.11. 2021**	**0**	**0**	**0**	**0**	**0**	**0**	**0**
**108**	**Marshall Islands**	**26.11. 2021**	**0**	**0**	**0**	**0**	**0**	**0**	**0**
**109**	**Mauritania**	**26.11. 2021**	**0**	**0**	**0**	**0**	**0**	**0**	**0**
**110**	**Mauritius**	**26.11. 2021**	**0**	**0**	**0**	**0**	**0**	**0**	**0**
**111**	**Mexico**	**26.11. 2021**	**9**	**1**	**8**	**7**	**1**	**1**	**0**
**112**	**Micronesia**	**26.11. 2021**	**0**	**0**	**0**	**0**	**0**	**0**	**0**
**113**	**Moldova**	**26.11. 2021**	**0**	**0**	**0**	**0**	**0**	**0**	**0**
**114**	**Monaco**	**26.11. 2021**	**0**	**0**	**0**	**0**	**0**	**0**	**0**
**115**	**Mongolia**	**26.11. 2021**	**5**	**2**	**3**	**1**	**2**	**1**	**1**
**116**	**Montenegro**	**26.11. 2021**	**1**	**0**	**1**	**1**	**0**	**0**	**0**
**117**	**Morocco**	**26.11. 2021**	**0**	**0**	**0**	**0**	**0**	**0**	**0**
**118**	**Mozambique**	**26.11. 2021**	**0**	**0**	**0**	**0**	**0**	**0**	**0**
**119**	**Myanmar (formerly Burma)**	**26.11. 2021**	**1**	**0**	**1**	**1**	**0**	**0**	**0**
**120**	**Namibia**	**26.11. 2021**	**0**	**0**	**0**	**0**	**0**	**0**	**0**
**121**	**Nauru**	**26.11. 2021**	**0**	**0**	**0**	**0**	**0**	**0**	**0**
**122**	**Nepal**	**26.11. 2021**	**3**	**1**	**2**	**2**	**0**	**0**	**0**
**123**	**Netherlands**	**26.11. 2021**	**50**	**7**	**43**	**38**	**5**	**2**	**3**
**124**	**New Zealand**	**26.11. 2021**	**17**	**3**	**14**	**14**	**0**	**0**	**0**
**125**	**Nicaragua**	**26.11. 2021**	**0**	**0**	**0**	**0**	**0**	**0**	**0**
**126**	**Niger**	**26.11. 2021**	**0**	**0**	**0**	**0**	**0**	**0**	**0**
**127**	**Nigeria**	**26.11. 2021**	**2**	**0**	**2**	**2**	**0**	**0**	**0**
**128**	**North Korea**	**26.11. 2021**	**0**	**0**	**0**	**0**	**0**	**0**	**0**

© 2022 Kuitunen I et al. *JAMA Network Open.*

129	North Macedonia	26.11. 2021	0	0	0	0	0	0	0
130	Norway	26.11. 2021	49	5	44	36	8	5	3
131	Oman	26.11. 2021	0	0	0	0	0	0	0
132	Pakistan	26.11. 2021	5	1	4	4	0	0	0
133	Palau	26.11. 2021	0	0	0	0	0	0	0
134	Palestine State	26.11. 2021	0	0	0	0	0	0	0
135	Panama	26.11. 2021	0	0	0	0	0	0	0
136	Papua New Guinea	26.11. 2021	0	0	0	0	0	0	0
137	Paraguay	26.11. 2021	0	0	0	0	0	0	0
138	Peru	26.11. 2021	0	0	0	0	0	0	0
139	Philippines	27.11. 2021	2	1	1	1	0	0	0
140	Poland	27.11. 2021	25	4	21	17	4	4	0
141	Portugal	27.11. 2021	7	1	6	6	0	0	0
142	Qatar	27.11. 2021	3	0	3	3	0	0	0
143	Romania	27.11. 2021	9	2	7	3	4	2	2
144	Russia	27.11. 2021	8	1	7	7	0	0	0
145	Rwanda	27.11. 2021	0	0	0	0	0	0	0
146	Saint Kitts and Nevis	27.11. 2021	0	0	0	0	0	0	0
147	Saint Lucia	27.11. 2021	0	0	0	0	0	0	0
148	Saint Vincent and the Grenadines	27.11. 2021	0	0	0	0	0	0	0
149	Samoa	27.11. 2021	0	0	0	0	0	0	0
150	San Marino	27.11. 2021	0	0	0	0	0	0	0
151	Sao Tome and Principe	27.11. 2021	0	0	0	0	0	0	0
152	Saudi Arabia	27.11. 2021	19	4	15	9	6	6	0
153	Senegal	27.11. 2021	0	0	0	0	0	0	0
154	Serbia	27.11. 2021	1	0	1	1	0	0	0

**155**	**Seychelles**	**27.11. 2021**	**0**	**0**	**0**	**0**	**0**	**0**	**0**
**156**	**Sierra Leone**	**27.11. 2021**	**0**	**0**	**0**	**0**	**0**	**0**	**0**
**157**	**Singapore**	**27.11. 2021**	**10**	**3**	**7**	**5**	**2**	**1**	**1**
**158**	**Slovakia**	**27.11. 2021**	**15**	**3**	**12**	**10**	**2**	**1**	**1**
**159**	**Slovenia**	**27.11. 2021**	**3**	**1**	**2**	**0**	**2**	**0**	**2**
**160**	**Solomon Islands**	**27.11. 2021**	**0**	**0**	**0**	**0**	**0**	**0**	**0**
**161**	**Somalia**	**27.11. 2021**	**0**	**0**	**0**	**0**	**0**	**0**	**0**
**162**	**South Africa**	**27.11. 2021**	**12**	**2**	**10**	**10**	**0**	**0**	**0**
**163**	**South Korea**	**27.11. 2021**	**11**	**1**	**10**	**10**	**0**	**0**	**0**
**164**	**South Sudan**	**27.11. 2021**	**0**	**0**	**0**	**0**	**0**	**0**	**0**
**165**	**Spain**	**27.11. 2021**	**29**	**1**	**28**	**25**	**3**	**3**	**0**
**166**	**Sri Lanka**	**27.11. 2021**	**0**	**0**	**0**	**0**	**0**	**0**	**0**
**167**	**Sudan**	**27.11. 2021**	**0**	**0**	**0**	**0**	**0**	**0**	**0**
**168**	**Suriname**	**27.11. 2021**	**0**	**0**	**0**	**0**	**0**	**0**	**0**
**169**	**Sweden**	**27.11. 2021**	**27**	**6**	**21**	**18**	**3**	**1**	**2**
**170**	**Switzerland**	**27.11. 2021**	**38**	**5**	**33**	**31**	**2**	**1**	**1**
**171**	**Syria**	**27.11. 2021**	**0**	**0**	**0**	**0**	**0**	**0**	**0**
**172**	**Tajikistan**	**27.11. 2021**	**0**	**0**	**0**	**0**	**0**	**0**	**0**
**173**	**Tanzania**	**27.11. 2021**	**0**	**0**	**0**	**0**	**0**	**0**	**0**
**174**	**Thailand**	**27.11. 2021**	**7**	**1**	**6**	**4**	**2**	**0**	**2**
**175**	**Timor-Leste**	**27.11. 2021**	**0**	**0**	**0**	**0**	**0**	**0**	**0**
**176**	**Togo**	**27.11. 2021**	**1**	**0**	**1**	**1**	**0**	**0**	**0**
**177**	**Tonga**	**27.11. 2021**	**0**	**0**	**0**	**0**	**0**	**0**	**0**
**178**	**Trinidad and Tobago**	**27.11. 2021**	**0**	**0**	**0**	**0**	**0**	**0**	**0**
**179**	**Tunisia**	**27.11. 2021**	**0**	**0**	**0**	**0**	**0**	**0**	**0**
**180**	**Turkey**	**27.11. 2021**	**65**	**7**	**58**	**49**	**9**	**5**	**4**
**181**	**Turkmenistan**	**27.11. 2021**	**0**	**0**	**0**	**0**	**0**	**0**	**0**

© 2022 Kuitunen I et al. *JAMA Network Open.*

**182**	**Tuvalu**	**27.11. 2021**	**0**	**0**	**0**	**0**	**0**	**0**	**0**
**183**	**Uganda**	**27.11. 2021**	**0**	**0**	**0**	**0**	**0**	**0**	**0**
**184**	**Ukraine**	**27.11. 2021**	**1**	**0**	**1**	**1**	**0**	**0**	**0**
**185**	**United Arab Emirates**	**27.11. 2021**	**3**	**1**	**2**	**0**	**2**	**1**	**1**
**186**	**United Kingdom**	**27.11. 2021**	**231**	**52**	**179**	**155**	**24**	**13**	**11**
**187**	**United States of America**	**27.11. 2021**	**349**	**38**	**311**	**304**	**7**	**5**	**2**
**188**	**Uruguay**	**27.11. 2021**	**1**	**0**	**1**	**1**	**0**	**0**	**0**
**189**	**Uzbekistan**	**27.11. 2021**	**0**	**0**	**0**	**0**	**0**	**0**	**0**
**190**	**Vanuatu**	**27.11. 2021**	**0**	**0**	**0**	**0**	**0**	**0**	**0**
**191**	**Venezuela**	**27.11. 2021**	**0**	**0**	**0**	**0**	**0**	**0**	**0**
**192**	**Vietnam**	**27.11. 2021**	**0**	**0**	**0**	**0**	**0**	**0**	**0**
**193**	**Yemen**	**27.11. 2021**	**0**	**0**	**0**	**0**	**0**	**0**	**0**
**194**	**Zambia**	**27.11. 2021**	**0**	**0**	**0**	**0**	**0**	**0**	**0**
**195**	**Zimbabwe**	**27.11. 2021**	**2**	**1**	**1**	**1**	**0**	**0**	**0**
	**TOTAL**		**1899**	**270**	**1629**	**1426**	**203**	**127**	**76**

© 2022 Kuitunen I et al. *JAMA Network Open.*

eTable 2. Background Characteristics of the Included Studies

	N	%
Screening method		
Clinical	15	19
Selective US	29	39
Universal US	32	42
Geographical location		
Europe	45	59
Asia	21	28
Australia and Oceania	6	8
North America	3	4
South America	1	1
Africa	0	0
Study design		
Register	16	21
Hospital	60	79
Follow-up		
Prospective	33	43
Retrospective	43	57

© 2022 Kuitunen I et al. *JAMA Network Open.*

eTable 3. Risk of Bias Assessment of the Individual Studies Based on the Joanna Briggs Institute Critical Appraisal Tool for Prevalence Studies

#	1st author	year	Was the sample frame appropriate to address the target population?	Were study participants sampled in an appropriate way?	Was the sample size adequate?	Were the study subjects and the setting described in detail?	Was the data analysis conducted with sufficient coverage of the identified sample?	Were valid methods used for the identificatio n of the condition?	Was the condition measured in a standard, reliable way for all participants?	Was there appropriate statistical analysis?	Was the response rate adequate, and if not, was the low response rate managed appropriatel y?	Overall
1	Ang	1997	yes	yes	yes	yes	yes	yes	yes	yes	Not applicable	Include
2	Arti	2013	yes	yes	yes	yes	yes	yes	yes	yes	Not applicable	Include
3	Azzopardi	2017	yes	yes	yes	yes	yes	unclear	unclear	yes	Not applicable	Include
4	Bache	2003	yes	yes	yes	yes	yes	yes	yes	yes	Not applicable	Include
5	Barik	2021	yes	yes	yes	yes	yes	unclear	unclear	yes	Not applicable	Include
6	Bhalvani	2011	yes	yes	no	yes	yes	yes	yes	yes	Not applicable	Include
7	Bialik	1997	yes	yes	no	yes	yes	yes	yes	yes	Not applicable	Include
8	Bialik	1999	yes	yes	yes	yes	yes	yes	yes	yes	Not applicable	Include
9	Biedermann	2018	yes	yes	yes	yes	yes	yes	yes	yes	Not applicable	Include
10	Bjerkreim	1987	yes	yes	yes	yes	yes	yes	unclear	yes	Not applicable	Include
11	Boere-Boonekampi	1998	yes	yes	yes	yes	yes	yes	unclear	yes	Not applicable	Include
12	Broadhurst	2019	yes	yes	yes	yes	yes	unclear	unclear	yes	Not applicable	Include
13	Buonsenso	2021	yes	yes	yes	yes	yes	yes	yes	yes	Not applicable	Include
14	Burger	1990	yes	yes	yes	yes	yes	unclear	unclear	yes	Not applicable	Include
15	Cekic	2015	yes	yes	yes	yes	yes	yes	yes	yes	Not applicable	Include
16	Chotigavanichay a	2012	yes	yes	yes	unclear	yes	unclear	unclear	yes	Not applicable	Include

© 2022 Kuitunen I et al. *JAMA Network Open.*

17	Clarke	2012	yes	yes	yes	yes	yes	unclear	unclear	yes	Not applicable	Include
18	Colta	2016	yes	yes	yes	yes	yes	yes	yes	yes	Not applicable	Include
19	Degnan	2021	yes	yes	yes	yes	yes	yes	yes	yes	Not applicable	Include
20	De Martino	1994	yes	yes	yes	no	yes	yes	yes	yes	Not applicable	Include
21	Den	2021	yes	yes	yes	yes	yes	unclear	unclear	yes	Not applicable	Include
22	Donnelly	2015	yes	yes	yes	yes	yes	unclear	unclear	yes	Not applicable	Include
23	Geertsema	2018	yes	yes	no	yes	yes	yes	yes	yes	Not applicable	Include
24	Gharehdaghi	2011	yes	yes	yes	unclear	yes	yes	yes	yes	Not applicable	Include
25	Giannakopoulou	2002	yes	yes	yes	yes	yes	unclear	unclear	yes	Not applicable	Include
26	Goss	2002	yes	yes	yes	yes	yes	unclear	unclear	yes	Not applicable	Include
27	Güler	2016	yes	yes	yes	yes	yes	yes	yes	yes	Not applicable	Include
28	Gyurkovits	2019	yes	yes	yes	yes	yes	yes	yes	yes	Not applicable	Include
29	Hesaraki	2017	yes	yes	yes	yes	yes	unclear	unclear	yes	Not applicable	Include
30	Ishikawa	2008	yes	yes	yes	yes	yes	yes	yes	yes	Not applicable	Include
31	Kamath	2007	yes	yes	yes	yes	yes	unclear	unclear	yes	Not applicable	Include
32	Kokavec	2007	yes	yes	yes	yes	yes	yes	yes	yes	Not applicable	Include
33	Kolb	2015	yes	yes	yes	yes	yes	yes	yes	yes	Not applicable	Include
34	Krolo	2003	yes	yes	yes	yes	yes	yes	yes	yes	Not applicable	Include
35	Kumar	2016	yes	yes	yes	yes	yes	yes	unclear	yes	Not applicable	Include
36	Kural	2019	yes	yes	yes	yes	yes	yes	unclear	yes	Not applicable	Include
37	Kose	2006	yes	yes	yes	yes	yes	yes	yes	yes	Not applicable	Include
38	Lange	2017	yes	yes	yes	yes	yes	yes	yes	yes	Not applicable	Include

© 2022 Kuitunen I et al. *JAMA Network Open.*

39	Lisle	2012	yes	yes	yes	no	yes	unclear	unclear	yes	Not applicable	Include
40	Mamouri	2003	yes	yes	yes	yes	yes	unclear	unclear	yes	Not applicable	Include
41	Maxwell	2002	yes	yes	yes	yes	yes	unclear	unclear	yes	Not applicable	Include
42	McAllister	2018	yes	yes	yes	yes	yes	yes	unclear	yes	Not applicable	Include
43	Milligan	2020	yes	yes	yes	yes	yes	yes	yes	yes	Not applicable	Include
44	Moosa	2009	yes	yes	yes	yes	yes	unclear	unclear	yes	Not applicable	Include
45	Munkhuu	2013	yes	yes	yes	yes	yes	yes	yes	yes	Not applicable	Include
46	Muresan	2019	yes	yes	no	yes	yes	yes	yes	yes	Not applicable	Include
47	Olsen	2018	yes	yes	yes	yes	yes	yes	yes	yes	Not applicable	Include
48	Paton	2005	yes	yes	yes	yes	yes	yes	unclear	yes	Not applicable	Include
49	Peled	2008	yes	yes	yes	yes	yes	yes	yes	yes	Not applicable	Include
50	Peled	2008	yes	yes	yes	yes	yes	yes	yes	yes	Not applicable	Include
51	Phelan	2014	yes	yes	yes	yes	yes	unclear	unclear	yes	Not applicable	Include
52	Pollet	2016	yes	yes	yes	yes	yes	yes	unclear	yes	Not applicable	Include
53	Puol	1998	yes	yes	yes	yes	yes	yes	yes	yes	Not applicable	Include
54	Reidy	2019	yes	yes	yes	yes	yes	unclear	unclear	yes	Not applicable	Include
55	Riboni	2003	yes	yes	yes	yes	yes	yes	yes	yes	Not applicable	Include
56	Rosendahl	1996	yes	yes	yes	yes	yes	yes	yes	yes	Not applicable	Include
57	Schams	2017	yes	yes	yes	yes	yes	yes	yes	yes	Not applicable	Include
58	Sepulveda	2021	yes	yes	yes	yes	yes	unclear	unclear	yes	Not applicable	Include
59	Sharpe	2005	yes	yes	yes	yes	yes	unclear	unclear	yes	Not applicable	Include
60	Sirisabya	2019	yes	yes	yes	yes	yes	unclear	unclear	yes	Not applicable	Include

© 2022 Kuitunen I et al. *JAMA Network Open.*

61	Studer	2016	yes	yes	yes	yes	yes	unclear	unclear	yes	Not applicable	Include
62	Talbot	2017	yes	yes	yes	yes	yes	yes	unclear	yes	Not applicable	Include
63	Tong	2011	yes	yes	yes	unclear	yes	unclear	unclear	yes	Not applicable	Include
64	Treiber	2008	yes	yes	yes	yes	yes	yes	yes	yes	Not applicable	Include
65	Treiber	2021	yes	yes	yes	yes	yes	yes	yes	yes	Not applicable	Include
66	Tyagi	2016	yes	yes	yes	yes	yes	yes	unclear	yes	Not applicable	Include
67	Vafaee	2017	yes	yes	yes	yes	yes	yes	yes	yes	Not applicable	Include
68	Von Kries	2003	yes	yes	yes	yes	yes	unclear	unclear	yes	Not applicable	Include
69	Walter	1992	yes	yes	yes	yes	yes	yes	yes	yes	Not applicable	Include
70	Wenger	2013	yes	yes	yes	yes	yes	yes	unclear	yes	Not applicable	Include
71	Wenger	2019	yes	yes	yes	yes	yes	yes	yes	yes	Not applicable	Include
72	Wilf-Miron	2017	yes	yes	yes	yes	yes	yes	yes	yes	Not applicable	Include
73	Wirth	2004	yes	yes	yes	yes	yes	yes	unclear	yes	Not applicable	Include
74	Woodacre	2016	yes	yes	yes	yes	yes	yes	unclear	yes	Not applicable	Include
75	Yiv	1997	yes	yes	yes	yes	yes	yes	yes	yes	Not applicable	Include
76	Zenios	2000	yes	yes	yes	yes	yes	unclear	unclear	yes	Not applicable	Include

© 2022 Kuitunen I et al. *JAMA Network Open.*
