# Supplementary material for: Incidence of Neonatal Developmental Dysplasia of the Hip and Late Detection Rates Based on Screening Strategy: A Systematic Review and Meta-analysis
Source: JAMA Netw Open. 2022 Aug 18;5(8):e2227638. doi: 10.1001/jamanetworkopen.2022.27638 (PMC9389349; doi:10.1001/jamanetworkopen.2022.27638)
Supplement: Supplement 2. — Review Protocol [file jamanetwopen-e2227638-s002.pdf]

Universal ultrasound screening in the neonatal developmental dysplasia of the hip – a systematic review and meta-analysis

Study group:

Ilari Kuitunen MD, PhD; Department of Pediatrics, Mikkeli Central Hospital, Finland

Mikko Uimonen MD, PhD; Department of Surgery, Central Finland Hospital Nova, Finland

Marjut Haapanen MD, Institute of Clinical Medicine, University of Eastern Finland

Reijo Sund PhD, Professor; Institute of Clinical Medicine, University of Eastern Finland

Ilkka Helenius MD, PhD, Professor; Department of Orthopedics, Helsinki University Hospital

Ville Ponkilainen MD, PhD; Department of Surgery, Central Finland Hospital Nova, Finland

Review question: What is the global incidence of developmental dysplasia of the hip in neonates? Is universal ultrasound screening of hip more effective than clinical status or selective ultrasound in detecting developmental dysplasia of the hip in newborns and reduces the universal screening need for later surgical procedures.

Searches: Following databases will be used: PubMed (MEDLINE), Web of Science and Scopus. Following search term will be used in this review: (hip) AND (dysplasia) AND (incidence or epidemiolog*) AND (*country* for example australia or australian). Additional articles will be included if found in the references of included articles and assessed suitable to be included for review and analysis.

Types of study to be included: We will perform a systematic search for every country separately and analyze incidences of initial dysplasia diagnosis, initial treatment rate late and the incidence of late detected dysplasia cases and need for operation (closed or open). For this part we will include all observational studies presenting incidences and reporting population studied, finding and late detection rates + operation rates. If a study report retrospectively findings before and after starting of universal ultrasound, we will include only the numbers of the latest screening method used.

Condition or domain being studied: Developmental dysplasia of the hip means that the hip joint is not fully developed, and the ball and socket joint is loose, and the femoral head may dislocate temporarily or permanently from the acetabular cup. Clinically this can be diagnosed in Ortolan and Barlow maneuvers. Hip ultrasound can be used to evaluate the hip and measure angles. Based on the findings the severity can be classified. Some countries screen routinely all hips in hospital, while other countries screen all hips clinically and a later ultrasound examination is used. Developmental dysplasia can be found in up to 10% of ultrasounds in routine screening, whereas the clinical suspicion of dysplasia is around 1%.

Reason to conduct this review: Previous meta-analyses have not found additional value of universal ultrasound screening; however, the practice is still implemented in many countries and many studies have been published since the latest Cochrane review in 2013. Observational studies have reported benefits of screening as more dysplasia cases have been diagnosed. But the increased detection rate might not lead to improved health benefits. A concern is that these diagnoses will lead to overtreatment and increased healthcare costs without additional value to the patient.

Population: All neonates will be included.

Interventions: Universal ultrasound screening.

Control: Neonates with clinical evaluation but without routine ultrasound evaluation.

Main outcome measures: Initial detection rate of DDH. Initial treatment rates (harness or splinting). Late (more than 12 weeks) detections of DDH. Rate of surgically treated DDH. Cost-effectiveness of ultrasound screening, if reported.

Data extraction: Two authors will screen abstracts and full texts. Covidence software will be used in the screening and extracting process. Third party opinion will be asked in case of disagreement or alternatively agreement between the two screening authors will be sought. Two authors will perform data extraction independently. Following information will be extracted: authors, year of

publication, country where the study was conducted, study period, study design, original inclusion criteria, intervention and control, total number of people included in the study, and outcomes.

Risk of bias assessment: We will not conduct risk of bias assessment.

Strategy for data synthesis: The RevMan version 5.4 and R statistical software version 4.0.3 are used for the meta-analysis. Data analysis will be performed according to Cochrane handbook of systematic reviews guidelines. Pooled incidences will be calculated by metarate function in the Meta package in R. Forest plots will be presented. The inconsistency index statistic I^2 for heterogeneity will be calculated and if I^2 >40%, a random effect model is used. If I^2 <40%, a fixed effect model will be chosen.

Analysis of subgroups or subsets

None
